# Gβγ-SNAP25 exocytotic brake removal enhances insulin action, promotes adipocyte browning, and protects against diet-induced obesity

**DOI:** 10.1172/JCI160617

**Published:** 2023-10-02

**Authors:** Ryan P. Ceddia, Zack Zurawski, Analisa Thompson Gray, Feyisayo Adegboye, Ainsley McDonald-Boyer, Fubiao Shi, Dianxin Liu, Jose Maldonado, Jiesi Feng, Yulong Li, Simon Alford, Julio E. Ayala, Owen P. McGuinness, Sheila Collins, Heidi E. Hamm

**Affiliations:** 1 Department of Medicine, Division of Cardiovascular Medicine, Vanderbilt University Medical Center, Nashville, Tennessee, USA.; 2 Department of Pharmacology, Vanderbilt University, Nashville, Tennessee, USA.; 3 Department of Anatomy and Cell Biology, University of Illinois at Chicago, Chicago, Illinois, USA.; 4 Program in Chemical and Physical Biology, and; 5 Department of Molecular Physiology and Biophysics, Vanderbilt University, Nashville, Tennessee, USA.; 6 State Key Laboratory of Membrane Biology, Peking University School of Life Sciences, Beijing, China.; 7 PKU-IDG/McGovern Institute for Brain Research, Beijing, China.; 8 Peking-Tsinghua Center for Life Sciences, Academy for Advanced Interdisciplinary Studies, Peking University, Beijing, China.; 9 Chinese Institute for Brain Research, Beijing, China.

**Keywords:** Endocrinology, Metabolism, Adipose tissue, G protein&ndash;coupled receptors, Neuroendocrine regulation

## Abstract

Negative regulation of exocytosis from secretory cells is accomplished through inhibitory signals from G_i/o_ GPCRs by Gβγ subunit inhibition of 2 mechanisms: decreased calcium entry and direct interaction of Gβγ with soluble *N*-ethylmaleimide–sensitive factor attachment protein (SNAP) receptor (SNARE) plasma membrane fusion machinery. Previously, we disabled the second mechanism with a SNAP25 truncation (SNAP25Δ3) that decreased Gβγ affinity for the SNARE complex, leaving exocytotic fusion and modulation of calcium entry intact and removing GPCR-Gβγ inhibition of SNARE-mediated exocytosis. Here, we report substantial metabolic benefit in mice carrying this mutation. *Snap25*^Δ3/Δ3^ mice exhibited enhanced insulin sensitivity and beiging of white fat. Metabolic protection was amplified in *Snap25*^Δ3/Δ3^ mice challenged with a high-fat diet. Glucose homeostasis, whole-body insulin action, and insulin-mediated glucose uptake into white adipose tissue were improved along with resistance to diet-induced obesity. Metabolic protection in *Snap25*^Δ3/Δ3^ mice occurred without compromising the physiological response to fasting or cold. All metabolic phenotypes were reversed at thermoneutrality, suggesting that basal autonomic activity was required. Direct electrode stimulation of sympathetic neuron exocytosis from *Snap25*^Δ3/Δ3^ inguinal adipose depots resulted in enhanced and prolonged norepinephrine release. Thus, the Gβγ-SNARE interaction represents a cellular mechanism that deserves further exploration as an additional avenue for combating metabolic disease.

## Graphical abstract

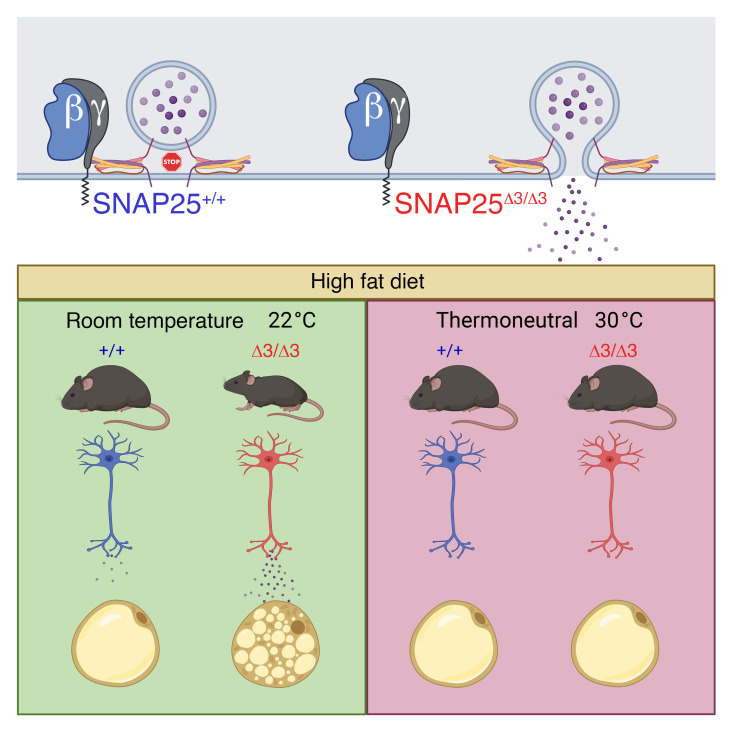

## Introduction

Metabolic diseases, such as diabetes and obesity, have an estimated annual global economic impact in the trillions of (US) dollars (1–5). Therapeutic approaches to lower metabolic risk include augmenting insulin secretion, limiting adiposity either by suppressing food intake or increasing energy expenditure, and/or by improving insulin action. GPCRs in metabolically important target tissues such as the liver, brain, adipose tissue, and pancreatic islets play critical roles in metabolic regulation and energy balance (6). Particularly in type 2 diabetes, pharmacologic agents have leveraged incretin-mediated GPCR signals to augment glucose-stimulated insulin secretion (GSIS) and attenuate weight gain (7). However, there may be additional approaches to manipulate GPCR signaling to treat metabolic disease.

GPCRs mediate downstream signaling events through the activation of heterotrimeric G proteins (e.g., Gα and Gβγ). Signaling events mediated by the classical Gα subunits are well known, and targeting either the receptor or the Gα-generated signal in specific target tissues has been successful in many spheres of pharmacology including metabolic disease (8, 9). By contrast, signaling via the Gβγ subunits has received less attention. These subunits differ from Gα in their physiologic roles, which include regulation of K^+^ and Ca^+2^ ion channels via their membrane localization (10, 11). One function of Gβγ is to transduce feedback signals that modulate or inhibit neurotransmitter and hormone secretion (12). This Gβγ mechanism curbs GSIS in the pancreatic β cell by either modulating calcium entry and/or by directly binding to the exocytotic fusion complex (13). In β cells, norepinephrine (NE) is thought to inhibit insulin exocytosis via the α_2_-adrenergic receptor (α_2_AR) through the interaction of Gβγ and soluble *N*-ethylmaleimide–sensitive factor attachment protein (SNAP) receptor (SNARE) proteins (13). We have shown in neurons that this inhibition requires Gβγ binding to the SNARE complex through the last 3 amino acids of SNAP25 (14–16). To specifically study the contribution of Gβγ to the exocytotic fusion step in vivo, we developed an allele of *Snap25* that encodes a protein lacking these last 3 amino acids (therefore called the SNAP25Δ3 protein) and created a mouse in which *Snap25Δ3* replaces *Snap25* on both alleles (16). We have called this mouse *Snap25*^Δ3/Δ3^. We previously showed that SNAP25Δ3 can form SNARE complexes that undergo calcium-synaptotagmin–mediated zippering and exocytosis identical to that of SNAP25, but its ability to interact with Gβγ and the resulting GPCR-mediated inhibitory effect on exocytosis are disabled (16, 17). Using the *Snap25*^Δ3/Δ3^ mouse, we previously demonstrated the importance of the Gβγ/SNARE pathway in several signaling processes, including stress responses and pain processing, long-term potentiation, and spatial memory (16, 18). However, the effects on metabolism and the response to diet-induced obesity (DIO) are unknown.

GPCR- and SNARE-dependent signals are involved in regulating metabolically important activities, such as feeding behavior, energy balance, insulin secretion, and thermoregulation (19–21). However, the specific roles that Gβγ-dependent signals play in regulating these physiologic responses are unknown. For example, when a palatable high-fat diet (HFD) is made available, it is unknown whether Gβγ-SNARE–dependent signals serve to limit or amplify weight gain or contribute to the glucose dysregulation and metabolic inflexibility commonly seen in metabolic disease (22, 23). As Gβγ-SNARE–dependent signals can serve as physiological brakes, they may restrain insulin secretion, impair insulin action, limit satiety, or attenuate energy expenditure, thus aggravating adiposity and impairing glucose homeostasis. We hypothesized that attenuation of this physiological brake using the *Snap25*^Δ3/Δ3^ mouse would improve glucose homeostasis and improve metabolic disease phenotypes in response to a palatable HFD. Our data described here demonstrate that chow-fed *Snap25*^Δ3/Δ3^ mice were more insulin sensitive and displayed increased adipose tissue beiging. Moreover, in response to a HFD challenge, the removal of this Gβγ-SNARE braking mechanism attenuated adiposity, improved insulin action, and amplified sympathetic activity and subsequent browning of adipose tissue. Reducing sympathetic tone by placing animals in a thermoneutral environment removed the metabolic protection afforded by this Gβγ-SNARE braking mechanism. The resistance to DIO and improved glucose homeostasis when this braking mechanism was disabled suggest that the Gβγ-SNARE interaction is a physiologically important mechanism that may be leveraged to treat metabolic disease.

## Results

### Normal growth curves with increased white adipose tissue beiging in chow-fed Snap25^Δ3/Δ3^ mice.

**Figure 1:**
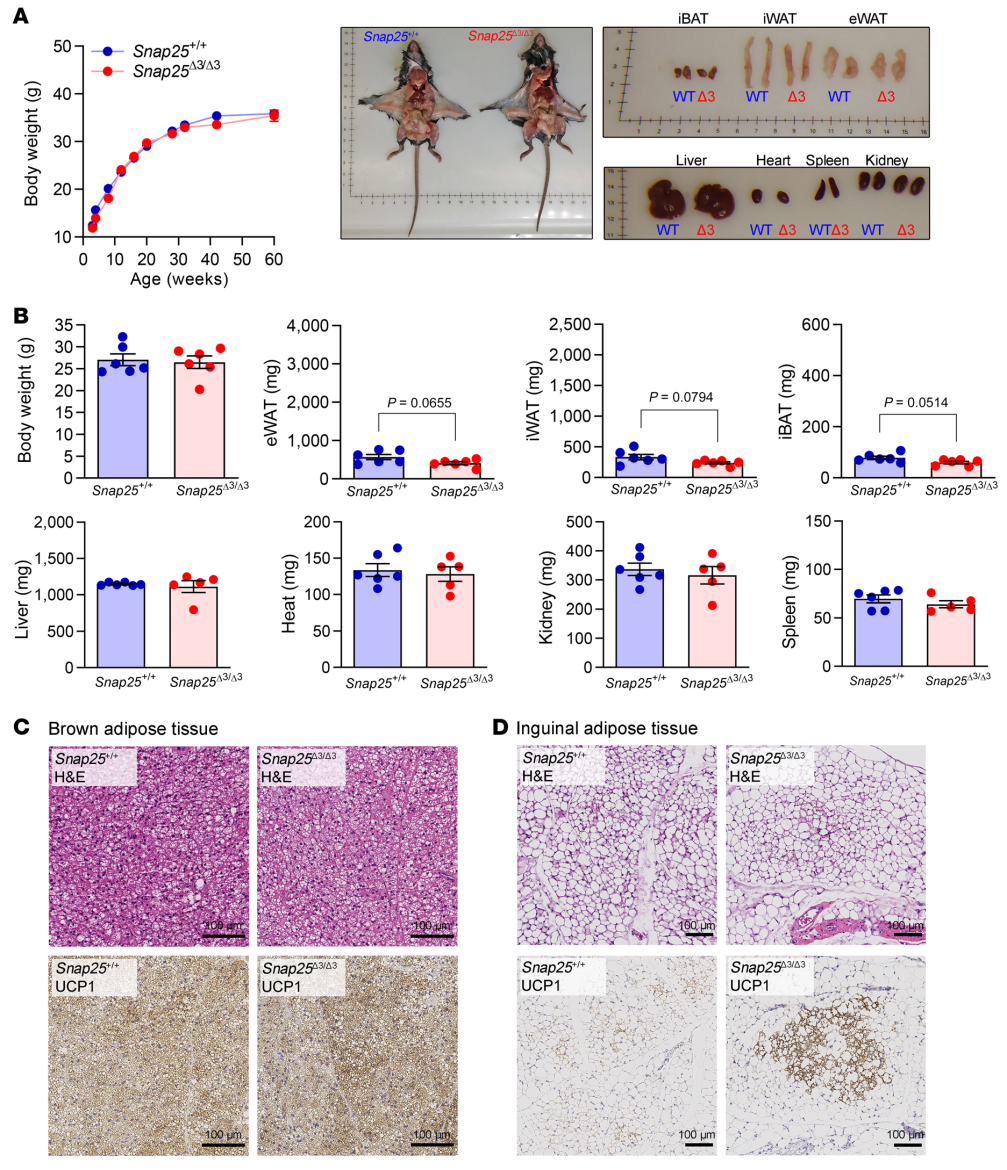
Body and tissue weights are unchanged, while WAT beiging is increased in chow-fed male *Snap25*^Δ3/Δ3^ mice. (**A**) Body weights of *Snap25*^+/+^ and *Snap25*^Δ3/Δ3^ male mice fed a standard chow diet (*P* = 0.3849, genotype; *P* = 0.0820, genotype × time interaction). *n* = 11 *Snap25*^+/+^ mice; *n* =11 *Snap25*^Δ3/Δ3^ mice. Analysis was performed by 2-way, repeated-measures ANOVA, and post hoc analyses were performed using Bonferroni’s multiple-comparison test for the *Snap25* genotype only. (**B**) Body and tissue weights of a separate cohort of chow-fed mice that were euthanized at 15 weeks of age (pictured). *n* = 6 *Snap25*^+/+^mice; *n* = 6 *Snap25*^Δ3/Δ3^ mice. Analyses were performed using an unpaired, 2-tailed Student’s *t* test. (**C**) Representative H&E- and UCP1-stained sections of iBAT. Scale bars: 100 μm. (**D**) Representative H&E- and UCP1-stained sections of iWAT. Scale bars: 100 μm. Images are a representative sample from 3 mice from each group. Values are the mean ± SEM.

Body weights of male and female *Snap25*^Δ3/Δ3^ mice were monitored over 60 weeks during which they were fed a standard chow diet; no differences were observed in growth curves compared with their *Snap25*^+/+^ littermates (Figure 1A and Supplemental Figure 1A; supplemental material available online with this article; https://doi.org/10.1172/JCI160617DS1). Internal organs and tissues weights were measured in a second cohort of 15-week-old mice, and no statistically significant difference was observed in most organ weights. As there was a trend toward slightly smaller adipose depots in *Snap25*^Δ3/Δ3^ mice (Figure 1B), we examined adipose histology. We observed a modest increase in UCP1-positive beige adipocytes in the inguinal white adipose tissue (iWAT) depot without an accompanying change in the interscapular brown adipose tissue (iBAT) (Figure 1, C and D). Consistent with a reduction in adipose tissue mass, we observed a decrease in the amount of circulating leptin (3.01 ± 0.46 vs. 1.24 ± 0.27 ng/mL leptin; *n* = 9 *Snap25*^+/+^ vs. 12 *Snap25*^Δ3/Δ3^ male mice; mean ± SEM; *P* = 0.002). These differences in adipose weight and remodeling were more evident in female mice (Supplemental Figure 1). A cohort of 15-week-old *Snap25*^Δ3/Δ3^ female mice weighed slightly less than the *Snap25*^+/+^ mice (Supplemental Figure 1B), had smaller fat pads (Supplemental Figure 1C), and reduced adipocyte size in both iBAT and iWAT (Supplemental Figure 1, D and E). Although it lacked a strong effect on the overall size and appearance of the mice, loss of the Gβγ-SNARE interaction led to a reduction in the size of adipocytes and their fat pad depots and was associated with increased WAT beiging.

### Lower glucose-stimulated insulin secretion in vivo without defects in glucose regulation suggests improved insulin action in Snap25^Δ3/Δ3^ mice fed a chow diet.

**Figure 2:**
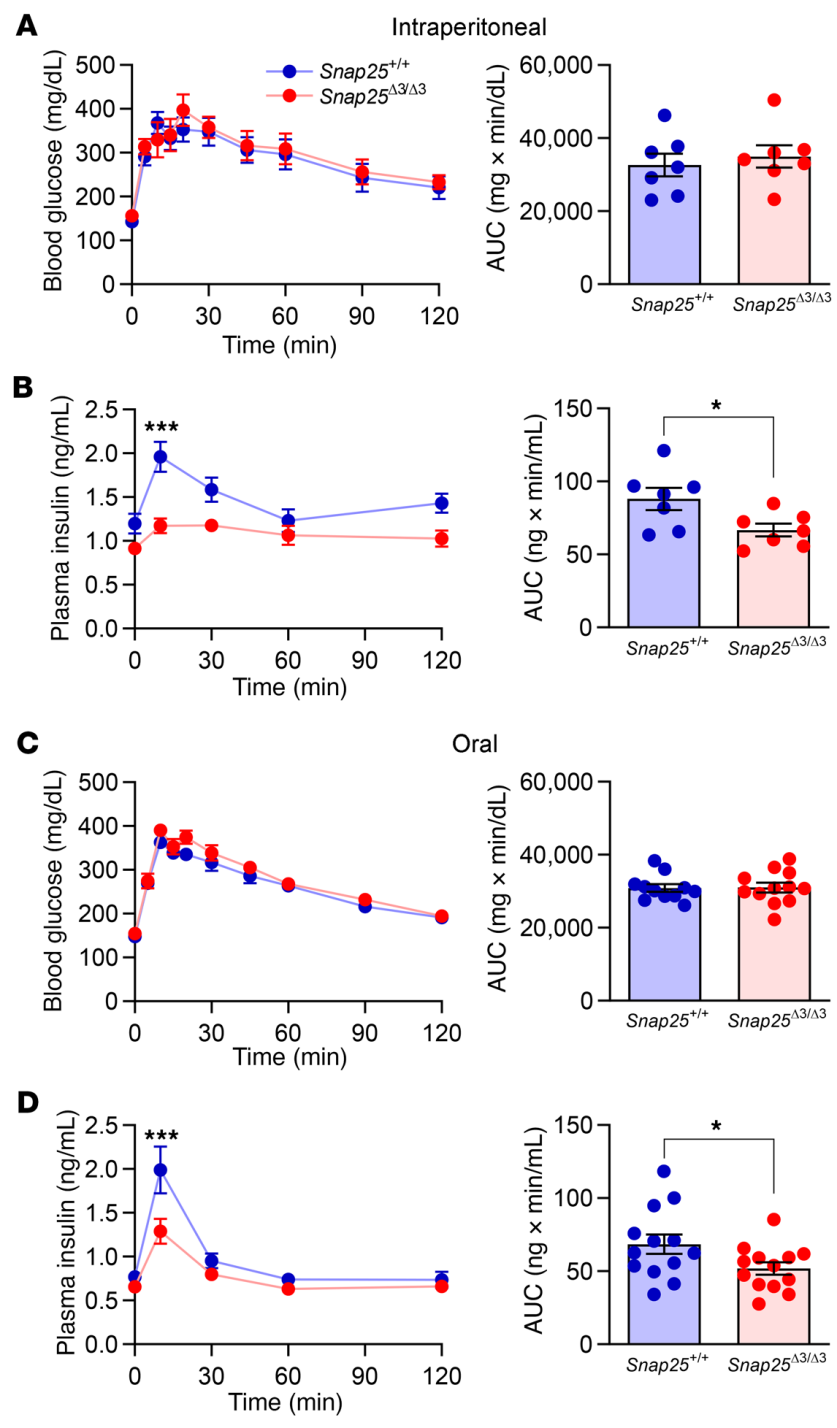
Glucose tolerance is normal but circulating insulin is reduced in chow-fed *Snap25*^Δ3/Δ3^ mice. (**A**) Blood glucose and (**B**) plasma insulin levels (*P* = 0.0176, genotype; *P* = 0.0031, genotype × time interaction) during an IP-GTT in chow-fed male *Snap25*^+/+^ and *Snap25*^Δ3/Δ3^ mice at 14 weeks of age. *n* = 8 *Snap25*^+/+^mice; *n* = 8 *Snap25*^Δ3/Δ3^ mice. (**C**) Blood glucose and (**D**) plasma insulin levels (*P* = 0.0730, genotype; *P* = 0.0009, genotype × time interaction) during an oral GTT in chow-fed male *Snap25*^+/+^ and *Snap25*^Δ3/Δ3^ mice at 15 weeks of age. *n* = 13 *Snap25*^+/+^ mice; *n* = 13 *Snap25*^Δ3/Δ3^ mice. Values are the mean ± SEM. *P < 0.05 and ***P < 0.001. For the glucose and insulin time course analysis, 2-way ANOVAs were performed with repeated measures, and post hoc analyses were performed using Bonferroni’s multiple-comparison test for the *Snap25* genotype only. AUCs were analyzed using an unpaired, 2-tailed Student’s *t* test.

To determine whether disabling the Gβγ inhibition of SNARE-mediated exocytosis would improve glucose homeostasis in chow-fed mice, we measured circulating glucose and insulin levels during an intraperitoneal glucose tolerance test in 14-week-old chow-fed mice. Glucose tolerance in the *Snap25*^Δ3/Δ3^ mice was not different from that of *Snap25*^+/+^ mice (Figure 2A). While basal insulin levels in *Snap25*^Δ3/Δ3^ mice were unaltered, insulin concentrations were reduced during the glucose tolerance test (GTT) compared with *Snap25*^+/+^ mice (Figure 2B), consistent with an improved insulin action. Since the incretin effect was bypassed with an intraperitoneal injection of glucose, we performed an oral GTT in a separate cohort of chow-fed mice to test whether this approach might unmask an augmentation of insulin secretion in the *Snap25*^Δ3/Δ3^ mice (24). Oral glucose tolerance was again not different between the genotypes (Figure 2C). However, as was observed with the intraperitoneal route of delivery, plasma insulin levels were lower in *Snap25*^Δ3/Δ3^ mice after the glucose gavage (Figure 2D). The lower levels of insulin with a normal glucose tolerance were consistent with improved insulin sensitivity in *Snap25*^Δ3/Δ3^ mice.

**Figure 3:**
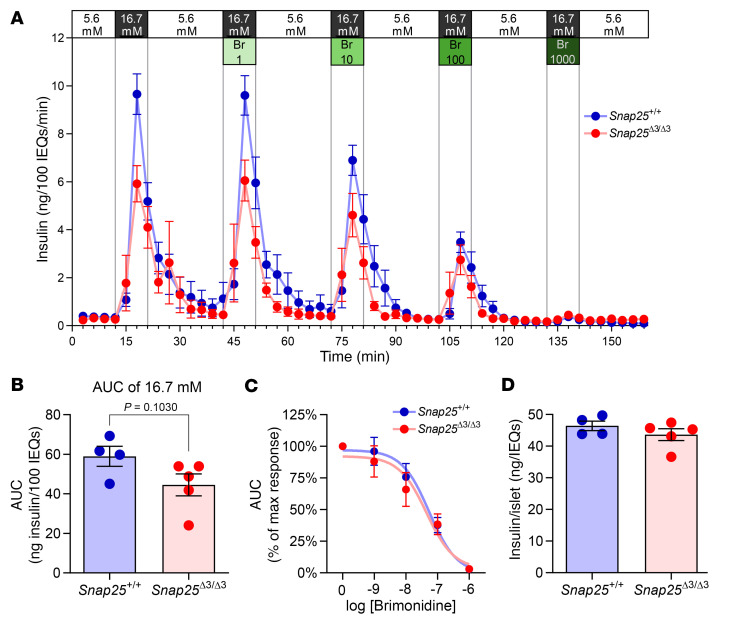
GSIS is not increased in islets from *Snap25*^Δ3/Δ3^ mice ex vivo, and inhibition by an α_2_AR agonist is not affected by disruption of the Gβγ-SNAP25 interaction. (**A**) Perifusion of islets from 12-week-old male *Snap25*^+/+^ and *Snap25*^Δ3/Δ3^ mice. (**B**) Comparison of GSIS between islets from *Snap25*^+/+^ and *Snap25*^Δ3/Δ3^ mice. (**C**) AUC values were normalized to the individual’s maximal GSIS, and a dose-response curve for the inhibition of GSIS by the α_2_AR-selective agonist Br was generated. The values for log IC_50_ were similar, with –7.262 for *Snap25*^+/+^ mice and –7.348 for *Snap25*^Δ3/Δ3^ mice. (**D**) Insulin content in islets from *Snap25*^+/+^ and *Snap25*^Δ3/Δ3^ mice. For all figures *n* = 4 *Snap25*^+/+^ mice; *n* = 5 *Snap25*^Δ3/Δ3^ mice. Values are the mean ± SEM. Analyses were performed using an unpaired, 2-tailed Student’s *t* test.

We observed no augmentation of insulin secretion during the GTT in vivo (Figure 2, B and D), although this could be explained by an accompanying improvement in insulin action. Other investigators reported in an immortalized mouse β cell line that inhibition of insulin exocytosis by NE via α_2_AR signaling was mediated by the Gβγ-SNARE interaction (13). We sought to determine in freshly isolated perifused islets if loss of the Gβγ-SNARE interaction improves GSIS and/or impairs α_2_AR-dependent inhibition of GSIS. We used a selective α_2_AR agonist, brimonidine (Br), to test this interaction. As shown in Figure 3, A and B, GSIS in isolated islets from *Snap25*^Δ3/Δ3^ mice was not greater than that from *Snap25*^+/+^ mice. Although islets from *Snap25*^Δ3/Δ3^ mice seemed to secrete less insulin, when multiple experiments were compiled there was no significant difference (Figure 3C). Moreover, contrary to the earlier studies in the cell line (13), Br was able to equally suppress GSIS in islets from *Snap25*^Δ3/Δ3^ and *Snap25*^+/+^ mice (Figure 3, A and C). The insulin content of the islets was also not significantly affected by the *Snap25* genotype (Figure 3D). This indicates that α_2_AR-mediated signaling in ex vivo mouse islets was able to inhibit GSIS via a mechanism that was independent of the Gβγ-SNARE interaction.

### Energy expenditure at room temperature and in response to cold does not differ between Snap25 genotype mice on a chow diet.

We assessed whether disabling the Gβγ-SNARE interaction alters the response to physiologic stressors, since we had previously reported that *Snap25*^Δ3/Δ3^ mice had an altered behavioral response to acute physiological stressors, specifically elevated stress-induced hyperthermia (16, 25). We performed calorimetric studies in which energy expenditure and feeding behavior in chow-fed *Snap25*^+/+^ and *Snap25*^Δ3/Δ3^ mice were measured at a standard housing temperature (22°C). The mice were then exposed to an acute cold challenge (6°C) to assess whether they could mount a physiologic response by increasing energy expenditure and food intake. At a normal housing temperature, *Snap25*^Δ3/Δ3^ mice had similar rates of energy expenditure (Supplemental Figure 2A) and food intake (Supplemental Figure 2B), with normal diurnal patterns for both variables. In response to a decrease in the environmental temperature, both *Snap25*^Δ3/Δ3^ mice and their *Snap25*^+/+^ littermates mounted a robust increase in energy expenditure. This was also accompanied by a greater increase in the duration of feeding activity in *Snap25*^Δ3/Δ3^ mice (Supplemental Figure 2E). Interestingly, any changes in autonomic activity that might occur were not global. For example, in a separate cohort, cardiovascular parameters were measured at standard housing temperature using echocardiography, and no differences were observed in the *Snap25*^Δ3/Δ3^ mice (Supplemental Figure 3). Thus, on a chow diet, *Snap25*^Δ3/Δ3^ mice displayed subtle changes in feeding behavior that overall did not affect the energy balance, and the physiological autonomic activity–dependent response to cold was also intact.

### Protection from HFD-induced obesity and adiposity in Snap25^Δ3/Δ3^ mice.

**Figure 4:**
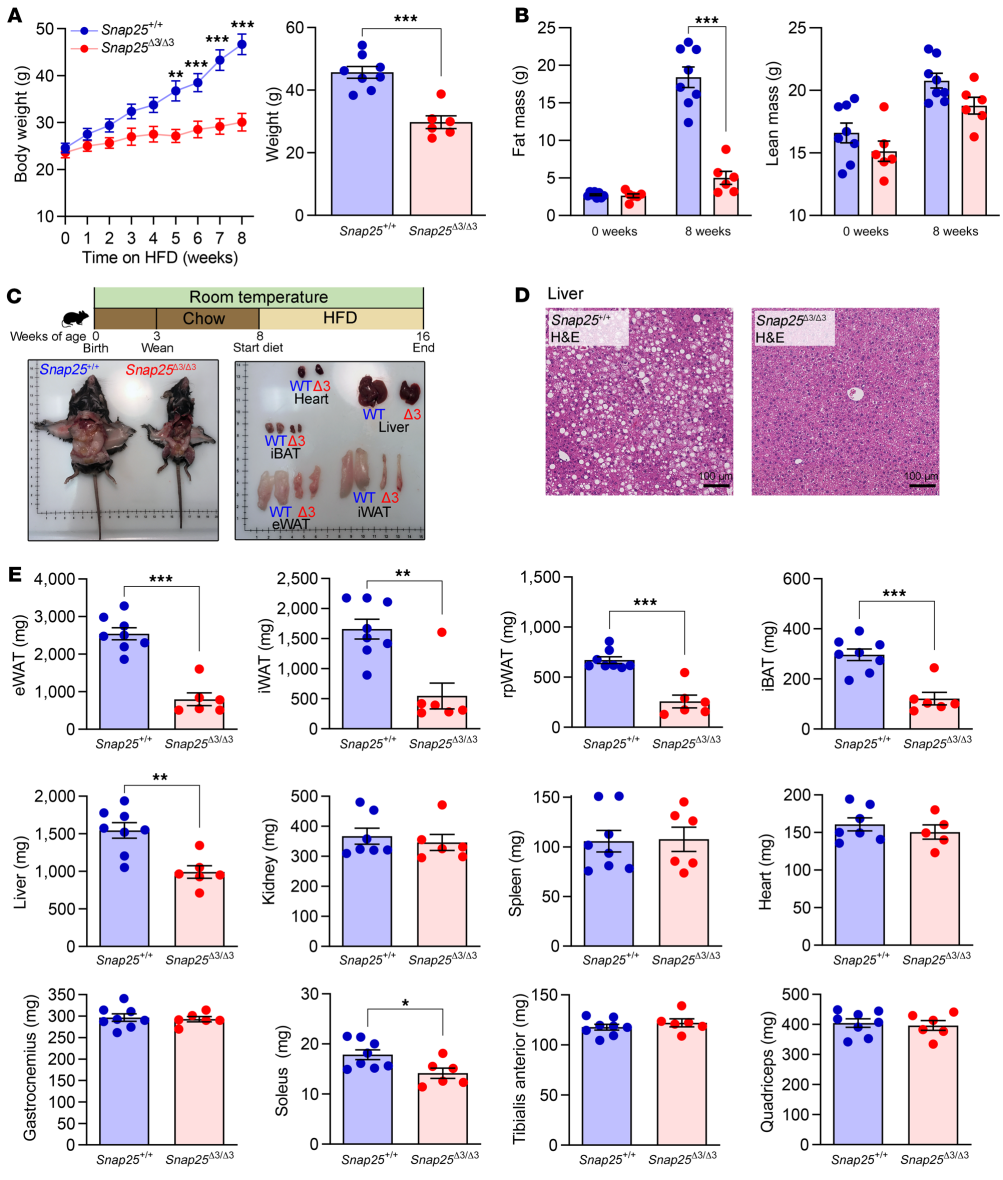
Weight gain and adiposity induced by a HFD are reduced in *Snap25*^Δ3/Δ3^ mice. (**A**) Male *Snap25*^+/+^ and *Snap25*^Δ3/Δ3^ mice were fed a HFD for 8 weeks beginning at 8 weeks of age (*P* = 0.0030, genotype; *P* < 0.001, genotype × time interaction). A comparison of terminal body weights is shown on the right. (**B**) Body composition of fat mass (*P* < 0.001, genotype; *P* < 0.001, genotype × time interaction) and lean mass (*P* = 0.0891, genotype) at the beginning and end of the HFD-feeding period. (**C**) Representative images of HFD-fed *Snap25*^+/+^ and *Snap25*^Δ3/Δ3^ mice and tissues postmortem. (**D**) Representative H&E-stained sections of livers. Images are a representative sample from 3 mice from each group. Scale bars: 100 μm. (**E**) Tissue weights of *Snap25*^+/+^ and *Snap25*^Δ3/Δ3^ mice at the end of the HFD-feeding period. For all panels, *n* = 8 *Snap25*^+/+^ mice; *n* = 6 *Snap25*^Δ3/Δ3^ mice. *P < 0.05, **P <0.01, and ***P <0.001. Values are the mean ± SEM. For **A** (left) and **B**, 2-way ANOVAs were performed with repeated measures, and post hoc analyses were performed using Bonferroni’s multiple-comparison test for the *Snap25* genotype only. Analyses for terminal body weight (**A** right) and **E** were performed using an unpaired, 2-tailed Student’s *t* test.

Given the apparent greater insulin sensitivity of the *Snap25*^Δ3/Δ3^ mice when on a chow diet (Figure 2), we tested whether this phenotype would persist when the mice were challenged with a HFD. We focused on male mice, given the robust improvement in insulin action observed in these mice on a chow diet. Eight-week-old *Snap25*^+/+^ and *Snap25*^Δ3/Δ3^ mice were provided a HFD for 8 weeks. As shown in Figure 4A, *Snap25*^+/+^ mice rapidly gained weight on the HFD, whereas *Snap25*^Δ3/Δ3^ mice were markedly resistant to HFD-induced weight gain. Body composition analyses revealed that the reduced weight gain in *Snap25*^Δ3/Δ3^ mice was due to reduced adiposity (Figure 4B) and was confirmed in postmortem analyses of these mice (Figure 4C). This was reflected in a decrease in plasma leptin levels (14.3 ± 1.32 vs. 3.53 ± 1.23 ng/mL leptin; *n* = 9 *Snap25*^+/+^ vs. 7 *Snap25*^Δ3/Δ3^; mean ± SEM; *P* < 0.001). In addition to a reduction in the weight of all fat depots (epididymal WAT [eWAT], iWAT, retroperitoneal WAT [rpWAT], and iBAT), liver weight and hepatic steatosis were also reduced in *Snap25*^Δ3/Δ3^ mice compared with *Snap25*^+/+^mice (Figure 4, D and E). The sizes of the kidney, spleen, heart, and most skeletal muscles were unaffected (Figure 4E).

### Improvement in insulin action in Snap25^Δ3/Δ3^ mice following a HFD challenge.

**Figure 5:**
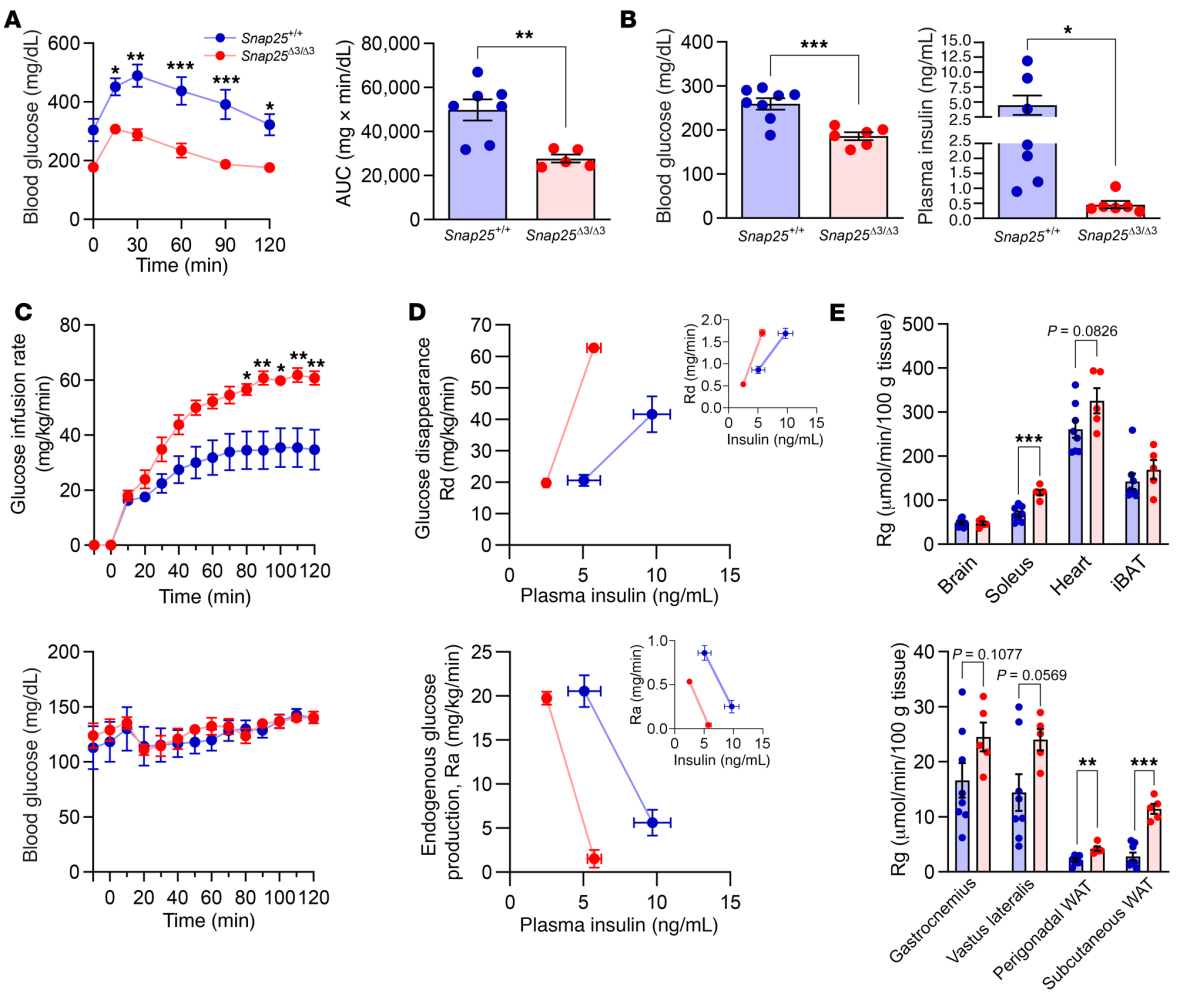
Insulin action and glucose homeostasis during a HFD challenge are improved in *Snap25*^Δ3/Δ3^ mice. (**A**) IP-GTT in male *Snap25*^+/+^ and *Snap25*^Δ3/Δ3^ mice fed a HFD for 8 weeks (*P* =0.0040, genotype; *P* = 0.0424, genotype × time interaction). *n* = 7 *Snap25*^+/+^mice; *n* = 5 *Snap25*^Δ3/Δ3^ mice. (**B**) Fasting blood glucose and plasma insulin levels at euthanasia. *n* = 7–8 *Snap25*^+/+^ mice; *n* = 6 *Snap25*^Δ3/Δ3^ mice. (**C**) Glucose infusion rate (*P* = 0.0224, genotype; *P* < 0.001, genotype × time interaction) and blood glucose levels in HFD-fed *Snap25*^+/+^ and *Snap25*^Δ3/Δ3^ male mice during the hyperinsulinemic euglycemic clamp procedure. (**D**) Glucose disappearance (Rd; mg/kg/min) and endogenous glucose production (Ra; mg/kg/min) plotted against plasma insulin levels. Insets show Ra and Rd (mg/min) not normalized to body weight, plotted against plasma insulin levels. (**E**) Tissue 2-DG uptake (Rg). For **C**–**E**, *n* = 8 *Snap25*^+/+^ mice; *n* = 5 *Snap25*^Δ3/Δ3^ mice. *P < 0.05, **P < 0.01, and ***P < 0.001. Values are the mean ± SEM. For **A**, **C**, and **D** (glucose and glucose infusion rate), a 2-way ANOVA was performed with repeated-measures, and post hoc analyses were performed using Bonferroni’s multiple-comparisons test for the *Snap25* genotype only. For **A** (AUC), **B** and **E**, analyses were analyzed using an unpaired, 2-tailed Student’s *t* test. Ra, rate of glucose appearance; Rd, rate of glucose disappearance.

Having observed a profound difference in weight gain and adiposity in *Snap25*^Δ3/Δ3^ mice when challenged with a HFD, we hypothesized that glucose homeostasis would also be improved. Indeed, HFD-fed *Snap25*^Δ3/Δ3^ mice showed a significant improvement in the intraperitoneal GTT (IP-GTT) compared with *Snap25*^+/+^ mice (Figure 5A). *Snap25*^Δ3/Δ3^ mice had lower 5-hour fasting glucose levels (Figure 5B), congruent with their lower IP-GTT curves. Plasma insulin levels were also significantly lower (Figure 5B). Together, these data indicate that there was a bona fide improvement in insulin sensitivity in the *Snap25*^Δ3/Δ3^ mice.

To determine the contribution of specific tissues to the improved glucose homeostasis of *Snap25*^Δ3/Δ3^ mice, we performed hyperinsulinemic euglycemic clamps in chronically catheterized conscious mice that had been consuming a HFD for 8 weeks (40.9 ± 2.5 vs. 27.2 ± 0.8 g body weight; *Snap25*^+/+^ vs. *Snap25*^Δ3/Δ3^; mean ± SEM). We determined whether insulin suppression of endogenous (i.e., hepatic) glucose production and/or stimulation of peripheral glucose uptake was altered. Arterial insulin levels were lower in the basal period in *Snap25*^Δ3/Δ3^ mice (5.8 ± 1.1 vs. 2.5 ± 0.2 ng/mL; *Snap25*^+/+^ vs. *Snap25*^Δ3/Δ3^; mean ± SEM) and increased with insulin infusion during the clamp period (9.7 ± 1.7 vs. 5.1 ± 0.8 ng/mL; *Snap25*^+/+^ vs. *Snap25*^Δ3/Δ3^; mean ± SEM). Arterial blood glucose was maintained at euglycemia in both groups throughout the clamp procedure (Figure 5C). The glucose infusion rate required to maintain euglycemia was increased in *Snap25*^Δ3/Δ3^ mice despite lower clamp insulin concentrations (Figure 5C). Using [3-^3^H]-glucose to assess whole-body glucose flux (mg/kg/min), we determined that the increase in glucose requirements in *Snap25*^Δ3/Δ3^ mice resulted from an increase in whole-body glucose uptake (Figure 5D). Basal endogenous glucose production as well as insulin suppression of endogenous glucose production were comparable, despite the fact that the rise in insulin was substantially lower in *Snap25*^Δ3/Δ3^ mice (Figure 5D). As *Snap25*^Δ3/Δ3^ mice were substantially leaner, we also calculated the glucose flux (mg/min) without dividing by body weight (Figure 5D, inset). Whole total glucose flux was not augmented during the clamp period (Figure 5D, inset), however, there was a shift to the left in the relationship between insulin suppression of glucose production as well as stimulation of whole-body glucose uptake. Thus, irrespective of how the data are presented, both hepatic and peripheral tissues of *Snap25*^Δ3/Δ3^ mice were more sensitive to insulin. To determine which tissues contributed to the increase in insulin action, we assessed the glucose metabolic index (Rg) in multiple tissues during the clamp using [^14^C]2-deoxy-d-glucose (2-DG). Rg was markedly increased in *Snap25*^Δ3/Δ3^ mice in multiple skeletal muscles (soleus and vastus lateralis) and WAT depots (perigonadal eWAT and subcutaneous iWAT) (Figure 5E). Rg was unchanged in iBAT, heart, gastrocnemius, and brain. Together, these data suggest that, despite much lower insulin levels during the clamp period, multiple tissues including the liver of *Snap25*^Δ3/Δ3^ mice were more responsive to insulin than that seen in *Snap25*^+/+^ mice.

### Elevation of tissue NE availability and increased beiging of WAT in Snap25^Δ3/Δ3^ mice following a HFD challenge.

**Figure 6:**
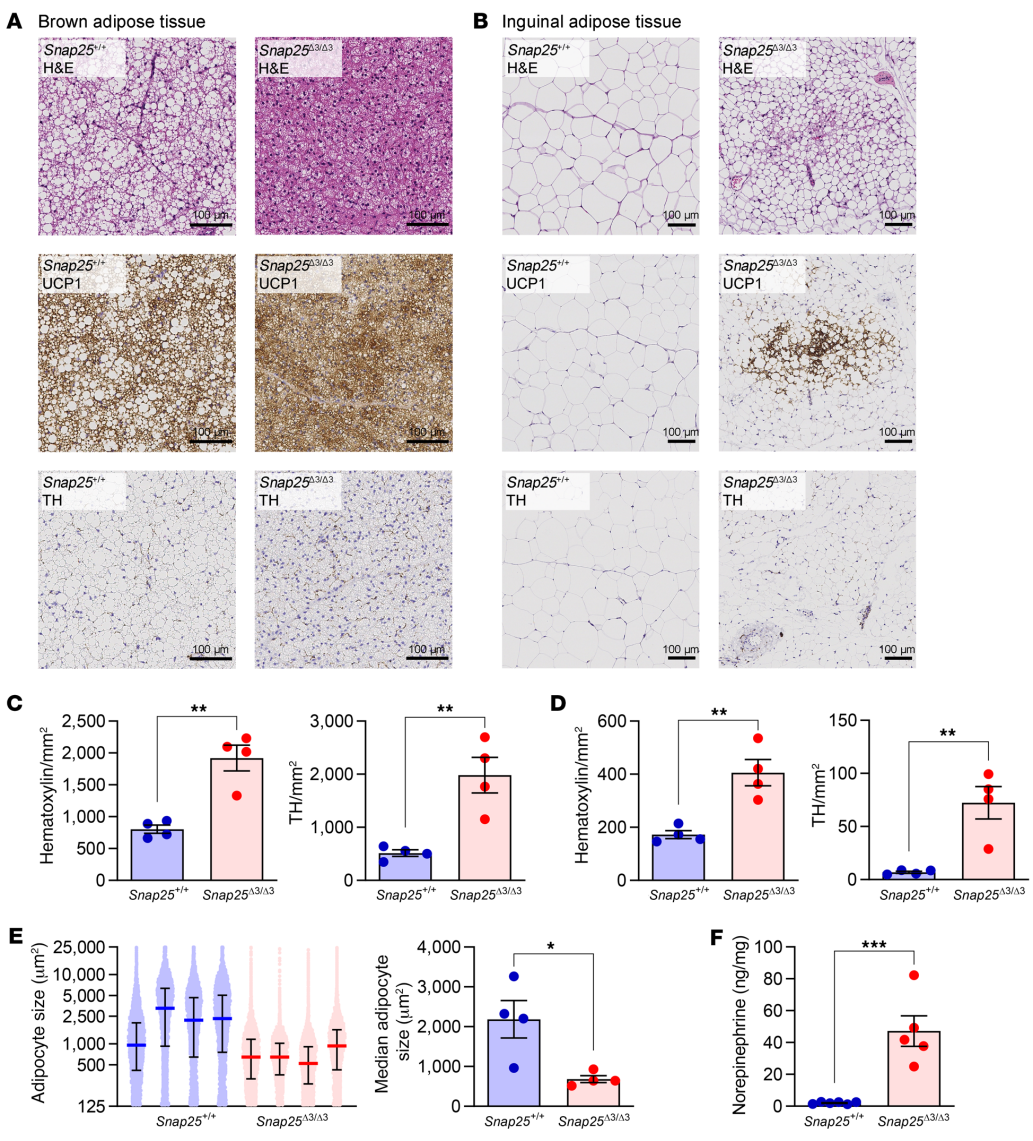
Adipose tissue remodeling in response to a HFD is replaced by increased NE-PKA signaling and beiging in *Snap25*^Δ3/Δ3^ mice. (**A**) Representative H&E-, UCP1-, and TH-stained sections of iBAT. Scale bars: 100 μm. (**B**) Representative H&E-, UCP1-, and TH-stained sections of iWAT. Scale bars: 100 μm. Images in **A** and **B** are representative samples from 3–4 mice from each group. (**C**) Quantification of nuclei (hematoxylin-stained) and TH-positive neurons in the iBAT TH-stained slides. (**D**) Quantification of nuclei (hematoxylin-stained) and TH-positive neurons in the iWAT TH-stained slides. (**E**) Quantification of iWAT adipocyte size in 4 mice of each genotype. On the left, each dot represents the area of an individual adipocyte, and each mouse is plotted separately. The median and IQR are plotted. On the right, the median iWAT adipocyte size for each mouse is plotted. (**F**) NE content in iWAT from HFD-fed *Snap25*^+/+^ and *Snap25*^Δ3/Δ3^ male mice. *n* = 6 *Snap25*^+/+^ mice; *n* = 5 *Snap25*^Δ3/Δ3^ mice. *P < 0.05, **P < 0.01, and ***P < 0.001. Values are the mean ± SEM. Analyses were performed using an unpaired, 2-tailed Student’s *t* test.

We then sought to investigate the changes that may have occurred in the adipose tissue of *Snap25*^Δ3/Δ3^ mice that could enhance their response to insulin and augment glucose uptake. In response to a HFD, *Snap25*^+/+^ mice developed normal remodeling and adipocyte hypertrophy of iBAT and iWAT (Figure 6, A–E). This contrasted with the *Snap25*^Δ3/Δ3^ mice, in which the median adipocyte size was significantly smaller in both iWAT and iBAT (Figure 6, A–E). The iBAT of HFD-fed *Snap25*^+/+^ mice had many large, unilocular adipocytes, whereas *Snap25*^Δ3/Δ3^ mice had smaller multilocular adipocytes (Figure 6A). F4/80 staining revealed that iWAT macrophages in *Snap25*^+/+^ mice were predominately located in crown-like structures but were more evenly distributed in *Snap25*^Δ3/Δ3^ mice (Supplemental Figure 4B). As has been observed in humans, increased beiging and the accompanying remodeling of the white adipose depots to be more oxidative may be responsible for some of the increased glucose uptake into WAT in *Snap25*^Δ3/Δ3^ mice (26). Indeed, as in the chow-fed mice, HFD-fed *Snap25*^Δ3/Δ3^ mice exhibited increased iWAT beiging (Figure 6B). Recent studies have shown an increase in the quantity of sympathetic nerve endings in beiged adipose tissue (27–32). Consistent with this finding, immunohistochemical staining of adipose tissue sections for tyrosine hydroxylase (TH), the first and rate-limiting enzyme in the catecholamine biosynthesis pathway, showed a significant increase in the number of TH-positive sympathetic innervations in both iBAT and iWAT (quantified in Figure 6, C an D). This increase in TH-positive nerves in the iWAT was evident in the stained sections (Figure 6, B and D).

The SNAP25Δ3 mutation prevents the Gβγ subunit from inhibiting vesicular membrane fusion and thereby the exocytosis of a currently undetermined number of neurotransmitters and hormones. There are a number of hormones that can elicit adipose tissue browning (6, 33). We sought to determine which hormones are altered, as this would shed light onto the mechanism behind the metabolic alterations in *Snap25*^Δ3/Δ3^ mice. The archetype signaling mechanism to induce adipocyte browning is cold, which stimulates the sympathetic nervous system to release the catecholamine NE into adipose tissue, which stimulates cAMP/PKA signaling events in adipocytes through the β-adrenergic receptors (βARs) (34). Because of this, we measured catecholamines by HPLC in these HFD-fed mice. Although circulating catecholamine levels were unchanged (Supplemental Figure 5), measurements directly from homogenized iWAT (2.06 ± 0.26 vs. 47.20 ± 9.61 ng/mg; *Snap25*^+/+^ vs. *Snap25*^Δ3/Δ3^; mean ± SEM) revealed a 23-fold increase in NE (Figure 6F). These data indicate that in *Snap25*^Δ3/Δ3^ mice, excess NE in the adipose tissues promoted beiging.

### Attenuation of the acute hyperphagic response to a HFD in Snap25^Δ3/Δ3^ mice.

**Figure 7:**
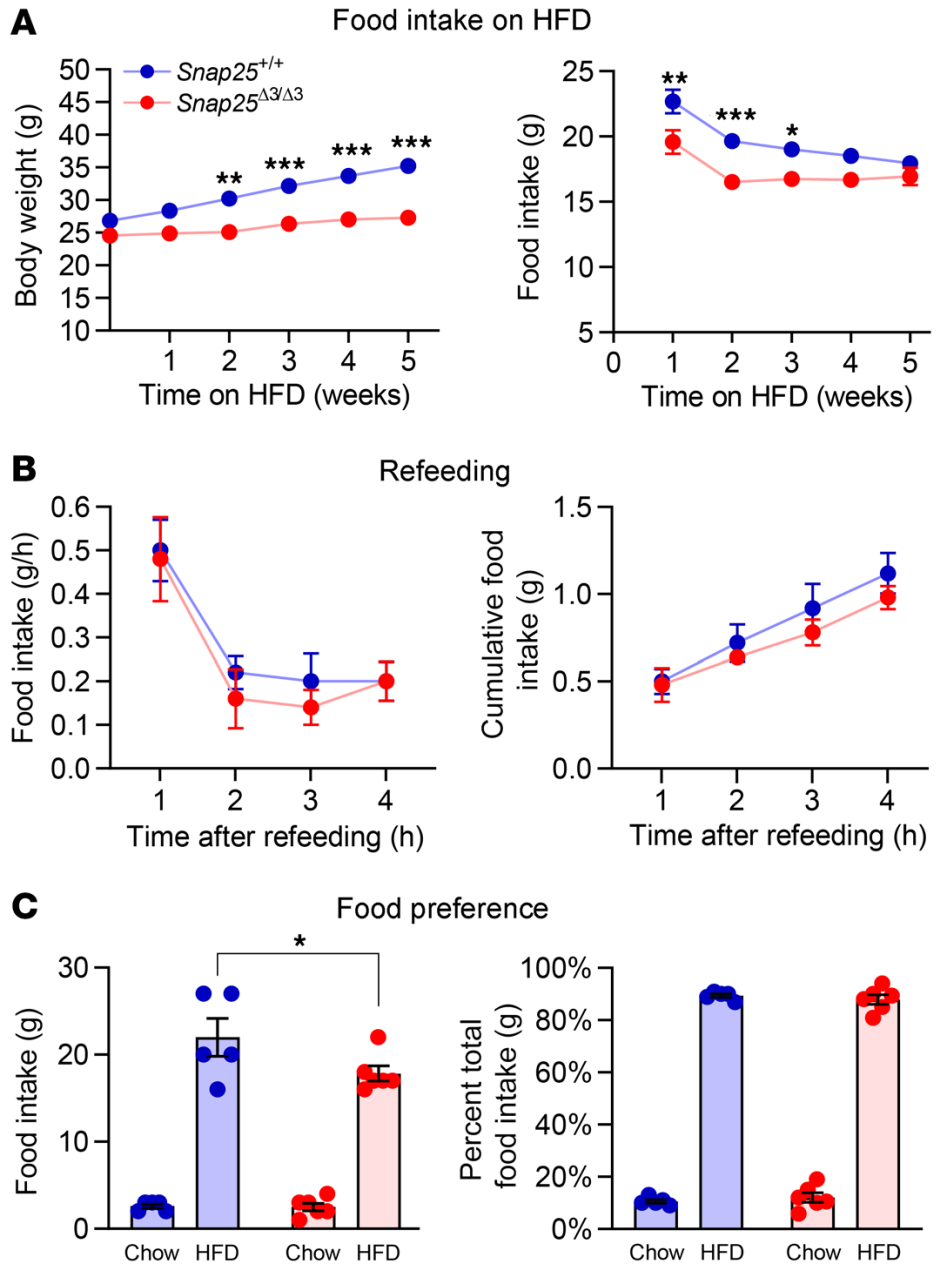
The initial hyperphagic response to a HFD is attenuated without differences in fasting-induced hyperphagia or dietary preference in *Snap25*^Δ3/Δ3^ mice. (**A**) Body weight (*P* < 0.001, genotype; *P* < 0.001, genotype × time interaction) and food consumption (*P* < 0.001, genotype; *P* = 0.2466, genotype × time interaction) during the first 5 weeks of HFD feeding in a separate cohort of male *Snap25*^+/+^ and *Snap25*^Δ3/Δ3^ mice. *n* = 23 *Snap25*^+/+^ mice; *n* = 18 *Snap25*^Δ3/Δ3^ mice. (**B**) Consumption of a HFD by male *Snap25*^+/+^ and *Snap25*^Δ3/Δ3^ mice following a 12-hour fast. *n* = 5 *Snap25*^+/+^ mice; *n* = 5 *Snap25*^Δ3/Δ3^ mice. (**C**) Cumulative food intake from chow-adapted male *Snap25*^+/+^ and *Snap25*^Δ3/Δ3^ mice given ad libitum access to both chow and a HFD. *n* = 5 *Snap25*^+/+^ mice; *n* = 6 *Snap25*^Δ3/Δ3^ mice. *P < 0.05, **P < 0.01, and ***P < 0.001. Values are the mean ± SEM. Analyses were performed using 2-way ANOVA, and post hoc analyses were performed using Bonferroni’s multiple-comparison test for the *Snap25* genotype only.

To determine the mechanism for the relative reduction in weight gain and fat mass in *Snap25*^Δ3/Δ3^ mice, we assessed lipid absorption, food intake, and energy expenditure in mice on a HFD. Fecal triglyceride content was not different (1.10 ± 0.29 vs. 0.86 ± 0.21 μg triglyceride/mg feces; *n* = 8 *Snap25*^+/+^ vs. *n* = 7 *Snap25*^Δ3/Δ3^ mice; mean ± SEM; *P* = 0.526), indicating that the reduction in body weights of *Snap25*^Δ3/Δ3^ mice was not due to a decrease in lipid absorption by the gut. We examined food intake over the first 5 weeks as chow-fed mice were switched to a HFD (Figure 7A). During the first 2 weeks of the HFD, *Snap25*^+/+^ mice exhibited a robust increase in food intake, which was attenuated in *Snap25*^Δ3/Δ3^ mice (Figure 7A). By week 4, the daily food consumption was similar between genotypes. To assess more empirically whether *Snap25*^Δ3/Δ3^ mice might be unable to mount a robust hyperphagic response, we used a fasting-refeeding paradigm, in which the animals were fasted for 12 hours and then presented with a HFD (Figure 7B). Mice of both genotypes had an equivalent and marked increase in HFD consumption over a 4-hour period. To assess food preference, we presented *Snap25*^Δ3/Δ3^ mice and their *Snap25*^+/+^ littermates with a HFD and chow in tandem and monitored total intake of both diets and body weight (Figure 7C). No significant differences were identified on the basis of genotype. In summary, we observed that *Snap25*^Δ3/Δ3^ mice did not overeat the more palatable, calorie-dense HFD to the same extent as their *Snap25*^+/+^ littermates, but this was not driven by an aversion to a HFD or the inability to mount a hyperphagic response.

**Figure 8:**
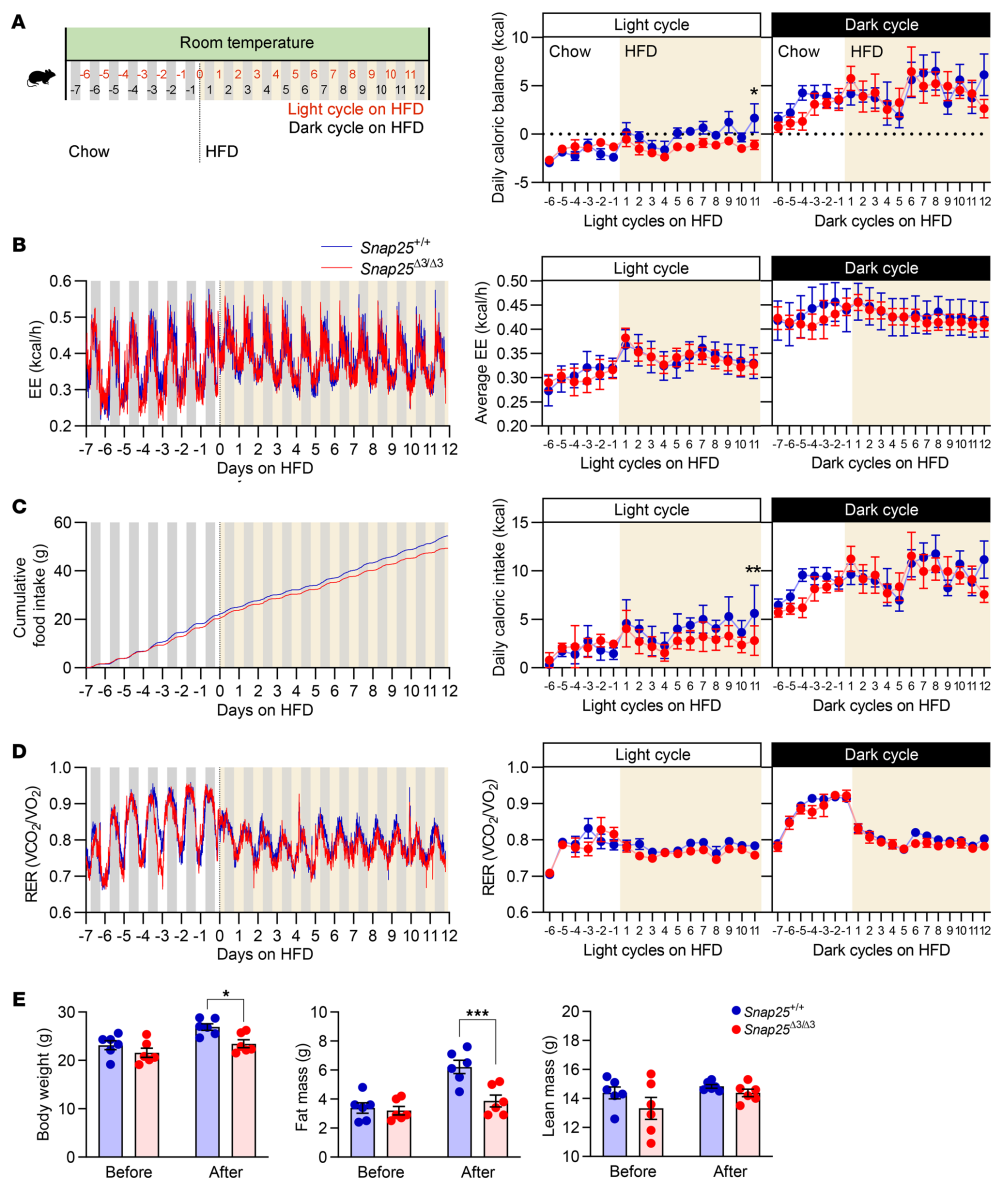
Lower net caloric accumulation upon transition to a HFD is associated with less food intake without alterations in energy expenditure in *Snap25*^Δ3/Δ3^ mice. (**A**) Chow-fed 12-week-old male *Snap25*^+/+^ and *Snap25*^Δ3/Δ3^ mice were acclimated in the Promethion System for 1 week prior to transitioning to a HFD. Graph shows the energy balance (*P* = 0.0006 genotype × time during the light cycle). (**B**) Energy expenditure (EE). (**C**) Cumulative food intake (*P* = 0.0556 genotype; *P* = 0.0023 genotype × time during the light cycle) and daily caloric intake. (**D**) Respiratory exchange ratio (RER). VO_2_, oxygen consumption volume; VCO_2_, CO_2_ production volume. (**E**) Body weights and composition before (day –7) and after (day 12) these energy balance studies. For all figures, *n* = 6 *Snap25*^+/+^ mice; *n* = 6 *Snap25*^Δ3/Δ3^ mice. *P < 0.05 and ***P < 0.001. Light and dark cycles were analyzed separately. Analyses were performed by 2-way ANOVA with repeated measures, and post hoc analyses were performed using Bonferroni’s multiple-comparison test for the *Snap25* genotype only. Energy expenditure was assessed using ANCOVA.

A more thorough assessment of this transition from chow to a HFD was performed using the Promethion calorimetry system to continuously monitor diurnal feeding patterns and energy expenditure (Figure 8A). We found that energy expenditure was not different and was highest in the dark phase in mice of both genotypes on a chow diet (Figure 8B). When presented with a HFD, mice of both genotypes showed a robust and comparable increase in energy expenditure and calorie intake (Figure 8, B and C). While energy expenditure and the respiratory exchange ratio did not differ between genotypes during the 12 days on a HFD (Figure 8, B and D). The initial hyperphagia gradually subsided in the *Snap25*^Δ3/Δ3^ mice to a greater extent than in the *Snap25*^+/+^ mice (Figure 8C). The 12-day cumulative calorie intake (Figure 8A), and, consequently, adiposity (Figure 8E), was decreased in *Snap25*^Δ3/Δ3^ mice. Thus, *Snap25*^Δ3/Δ3^ mice had a short-term but significantly (*P* = 0.0023) altered food consumption pattern that contributed to the reduced weight gain while on a HFD. Taken together, we can infer that altered feeding behavior, in particular the initial response to a HFD, in *Snap25*^Δ3/Δ3^ mice was a contributor to the differential weight-gain phenotype.

### Reversal of metabolic protection in Snap25^Δ3/Δ3^ mice by thermoneutral housing conditions.

**Figure 9:**
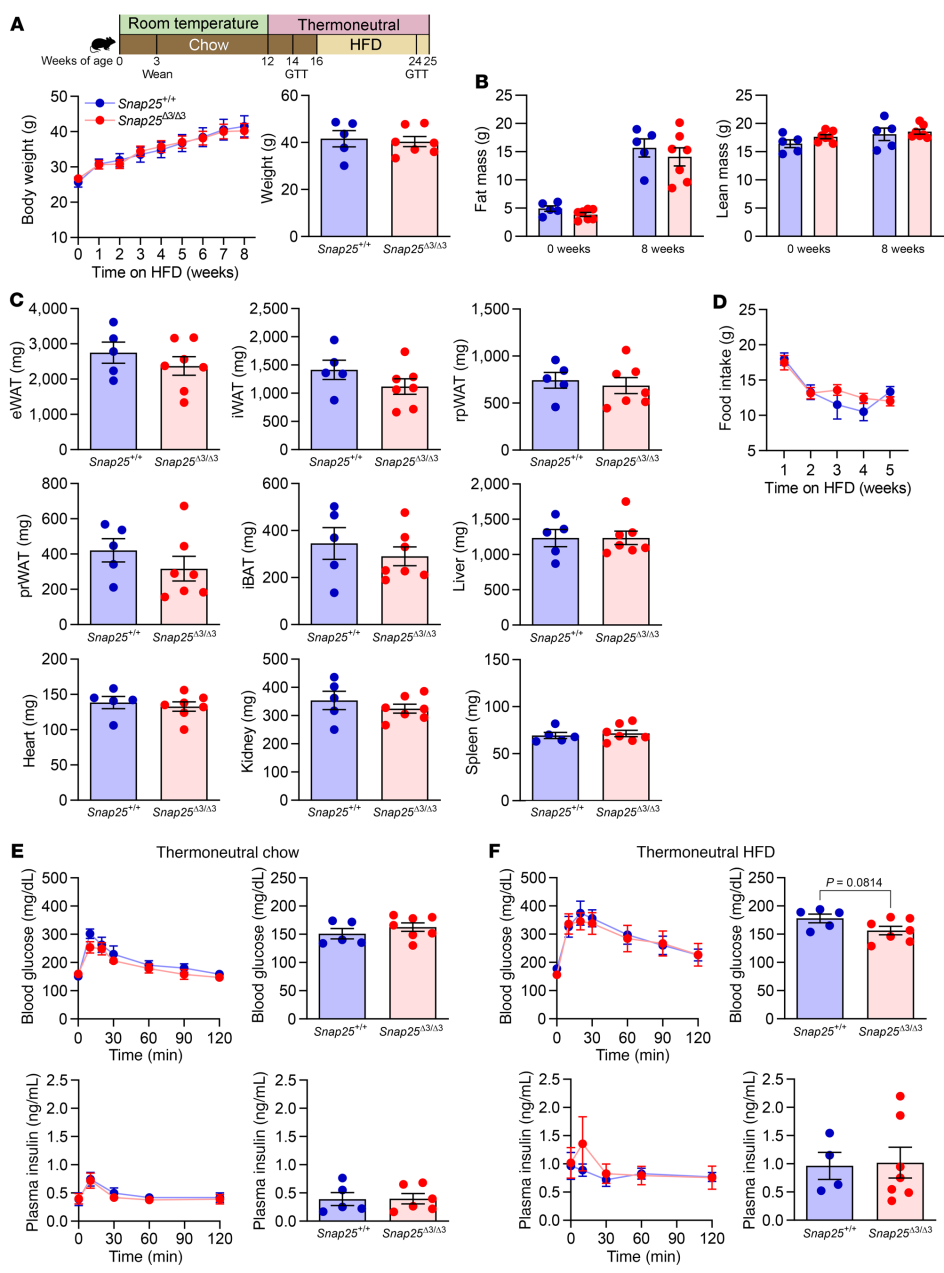
Lack of metabolic protection during HFD feeding in *Snap25*^Δ3/Δ3^ mice under thermoneutral housing conditions. (**A**) Following 4 weeks of acclimation to thermoneutral housing conditions, male *Snap25*^+/+^ and *Snap25*^Δ3/Δ3^ were fed a HFD for 8 weeks. Comparison of terminal body weights. (**B**) Body composition at the beginning and end of the HFD-feeding period. (**C**) Tissue weights for *Snap25*^+/+^ and *Snap25*^Δ3/Δ3^ mice at the end of the HFD-feeding period. (**D**) Food consumption during the first 5 weeks of HFD feeding during thermoneutral housing conditions. *n* = 5 *Snap25*^+/+^ mice; *n* = 7 *Snap25*^Δ3/Δ3^ mice. Two-way ANOVA with repeated measures and post hoc analyses were performed using Bonferroni’s multiple-comparison test for the *Snap25* genotype only. (**E**) Blood glucose and plasma insulin levels during an IP-GTT in chow-fed male *Snap25*^+/+^ and *Snap25*^Δ3/Δ3^ mice housed under thermoneutral conditions for 2 weeks. Fasting blood glucose and plasma insulin at “time 0” of the GTT. (**F**) Blood glucose and plasma insulin levels during an IP-GTT in HFD-fed male *Snap25*^+/+^ and *Snap25*^Δ3/Δ3^ mice housed under thermoneutral conditions. Fasting blood glucose and plasma insulin at “time 0” of the GTT. For all panels, *n* = 5 *Snap25*^+/+^ mice; *n* = 6–7 *Snap25*^Δ3/Δ3^ mice. Values are the mean ± SEM. Most analyses were performed by 2-way ANOVAs with repeated measures, and post hoc analyses were performed using Bonferroni’s multiple-comparison test for the *Snap25* genotype only. Exceptions were for terminal body weight (**A**, right), tissue weights (**C**), and fasting glucose and insulin (**D** and **E**, right), which were performed using an unpaired, 2-tailed Student’s *t* test.

A mild thermogenic stress was evoked in mice by housing them at standard room temperature (~22°C). This thermogenic stress leads to activation of the autonomic nervous system in adipose and other tissues to support thermoregulation (35). Increased autonomic drive and beiging of adipose tissue were observed in the *Snap25*^Δ3/Δ3^ mice kept in standard housing conditions (Figure 6), suggesting that the Gβγ-SNARE interaction was a negative regulator of autonomic activity. We hypothesized that if we minimized the induction of autonomic drive by shifting animals to a thermoneutral setting (~30°C), the metabolic benefits of *Snap25*^Δ3/Δ3^ would decrease. Therefore, we housed *Snap25*^Δ3/Δ3^ mice and their *Snap25*^+/+^ littermates in thermoneutral housing for 4 weeks prior to providing them a HFD. Indeed, the reduced weight gain, adiposity, and fat pad size of *Snap25*^Δ3/Δ3^ mice in response to a HFD were eliminated (Figure 9, A–C). Food intake in response to HFD feeding was also no longer different between genotypes during the thermoneutral housing conditions (Figure 9D). Thermoneutral housing did not revert food intake in *Snap25*^Δ3/Δ3^ mice to the amount seen in *Snap25*^+/+^ mice housed at room temperature; instead, in both genotypes, food intake was lower than for room temperature–housed animals during all weeks of the study (Figure 7A and Figure 9D). In addition, the improvements in the insulin action of *Snap25*^Δ3/Δ3^ mice during the IP-GTT at room temperature, in both the chow and HFD feeding periods, were also abolished by housing the mice in thermoneutral conditions (Figure 9, E and F). These findings demonstrate that the metabolic improvements caused by removing the Gβγ-SNARE interaction were dependent on the environmental temperature in which the mice were housed. This is consistent with our hypothesis that increased sympathetic activity due to loss of the α_2A_-adrenergic brake to exocytosis mediates the metabolic protection conferred to *Snap25*^Δ3/Δ3^ mice.

### Prevention of diet-induced remodeling of adipose tissues in Snap25^Δ3/Δ3^ mice by thermoneutral housing conditions.

**Figure 10:**
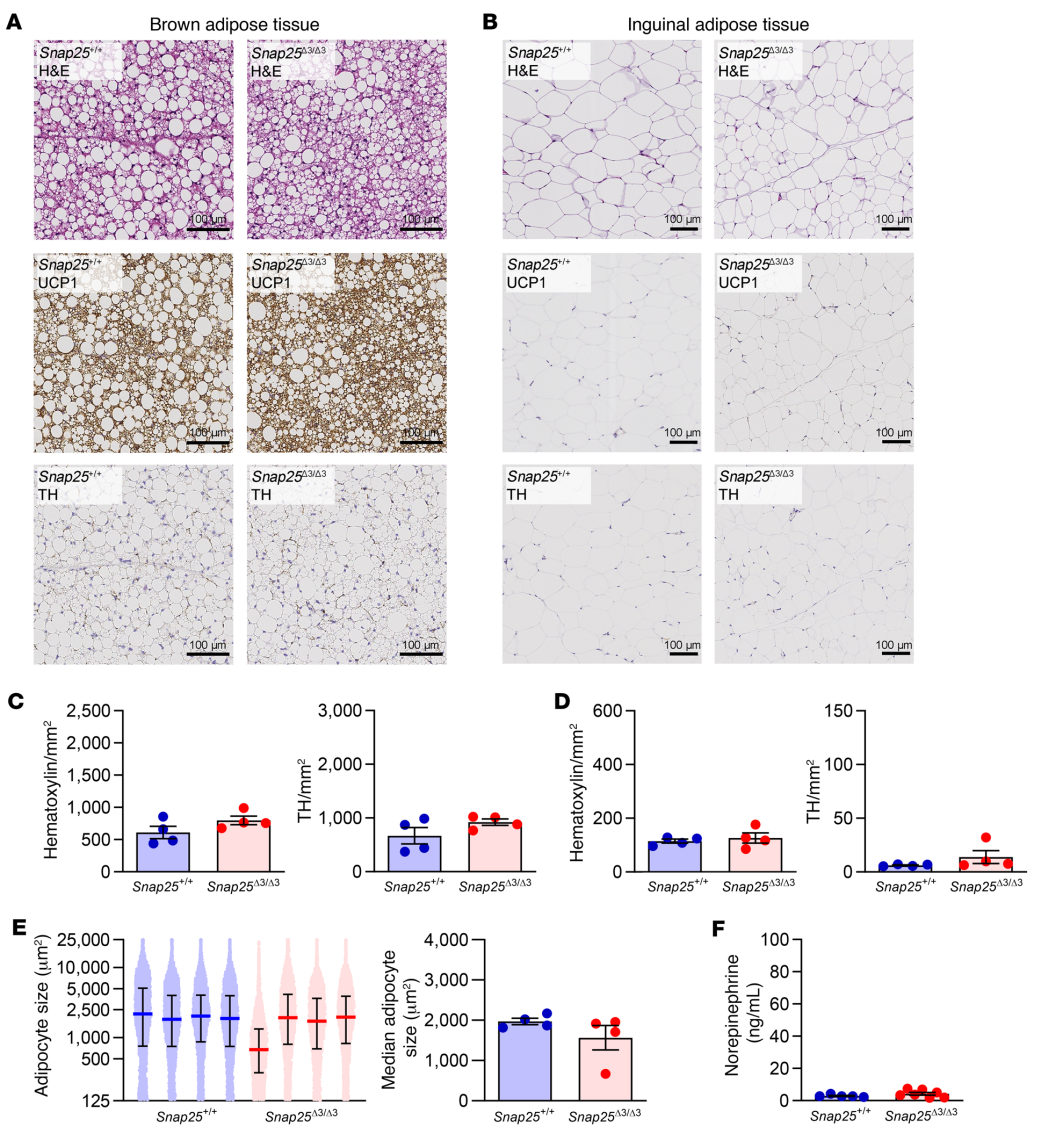
Adipose tissue remodeling in response to a HFD is not different in *Snap25*^Δ3/Δ3^ mice under thermoneutral housing conditions. (**A**) Representative H&E-, UCP1-, and TH-stained sections of iBAT. Scale bars: 100 μm. (**B**) Representative H&E-, UCP1-, and TH-stained sections of iWAT. Scale bars: 100 μm. (**C**) Quantification of nuclei (hematoxylin-stained) and TH-positive neurons in the iBAT TH-stained slides. (**D**) Quantification of nuclei (hematoxylin-stained) and TH-positive neurons in the iWAT TH-stained slides. Images are a representative sample from 3–4 mice from each group. (**E**) Quantification of iWAT adipocyte size in 4 mice of each genotype. On the left, each dot represents the area of an individual adipocyte, and each mouse is plotted separately. The median and IQR are plotted. On the right, the median iWAT adipocyte size for each mouse is plotted. (**F**) NE content in iWAT from HFD-fed *Snap25*^+/+^ and *Snap25*^Δ3/Δ3^ male mice. *n* = 5 *Snap25*^+/+^ mice; *n* = 7 *Snap25*^Δ3/Δ3^ mice. Values are the mean ± SEM. All analyses were performed using an unpaired, 2-tailed Student’s *t* test.

Increasing sympathetic activity to induce heat production from adipose tissue is a fundamental part of the response to environmental cold stress (36, 37). In the mice housed at thermoneutral conditions, this adipose beiging and elevation of NE content was eliminated (Figure 10, A–F). Both iBAT and iWAT from *Snap25*^Δ3/Δ3^ mice were remodeled as expected in response to HFD feeding (Figure 10, A–E), but no difference was seen between the genotypes. In mice of both genotypes, macrophages in iWAT were predominately located in crown-like structures (Supplemental Figure 4B). The ability of thermoneutral housing conditions to reverse the metabolic improvements achieved by disrupting the Gβγ-SNARE interaction indicates that these improvements were related to an elevation of autonomic activity in tissues such as adipose tissue.

### Release of NE from sympathetic endings is amplified in Snap25^Δ3/Δ3^ mice.

**Figure 11:**
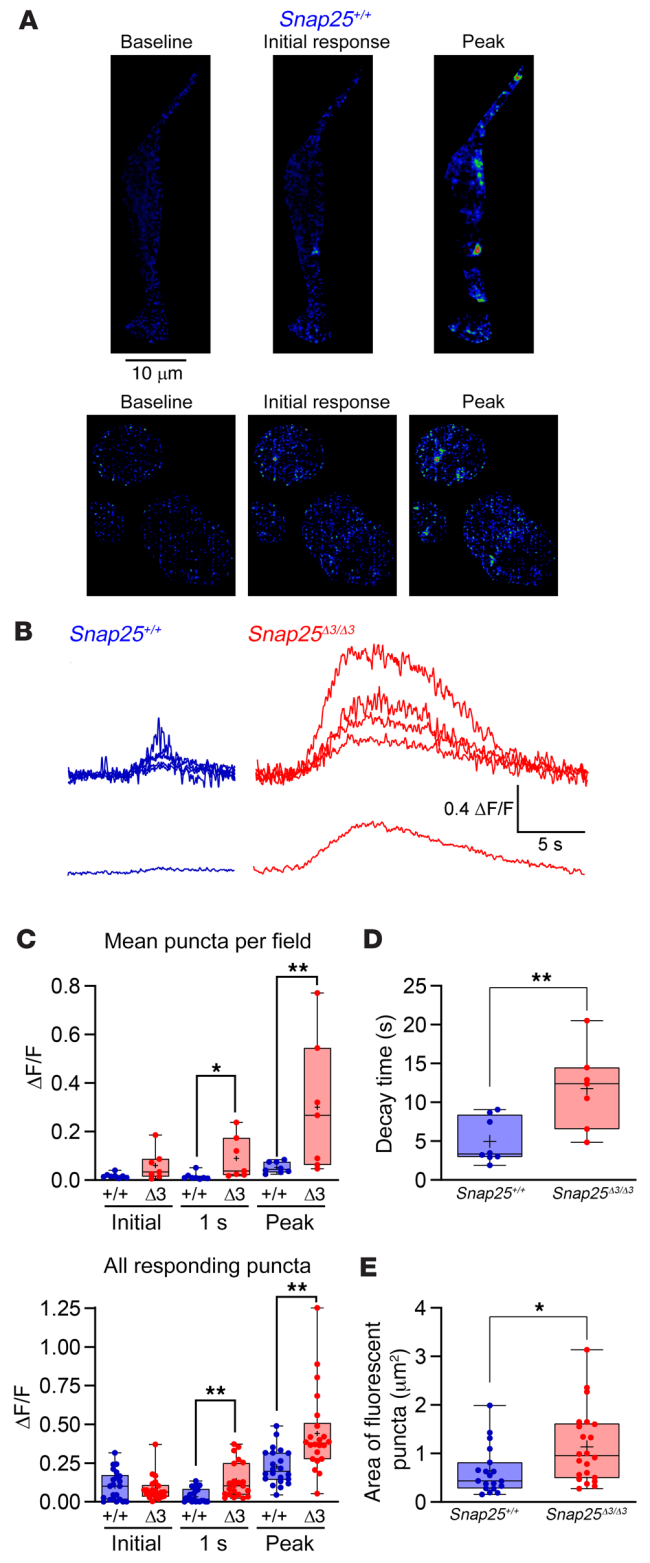
NE release measured ex vivo from sympathetic neurons in the inguinal adipose depot is greater in *Snap25*^Δ3/Δ3^ mice. (**A**) Live-cell images of 535 nm GRAB_NE_ fluorescence in mice hemizygous for Rosa26-LSL-GRAB-NE and *TH*-Cre showing fluorescent puncta surrounding, but not within, adipocytes before, during, and after stimulus trains of 100 stimuli, 100–200 μA at 20 Hz, and 1 ms duration. Scale bar: 10 μm. Areas of low fluorescence are depicted in blue, and areas of high fluorescence are depicted in red in the false-color spatial heatmap. (**B**) Representative fluorescence transients graphed as change in fluorescence intensity (ΔF/F) from iWAT *Snap25*^+/+^ mice (blue) and *Snap25*^Δ3/Δ3^ littermates (red). (**C**) Top: Bar graphs of mean ΔF/F values per field for iWAT taken from *Snap25*^+/+^ and *Snap25*^Δ3/Δ3^ immediately upon stimulation, 1 second after the stimulus, and at the peak of the stimulus. Bottom: Bar graphs of individual ΔF/F values per responding puncta for iWAT from *Snap25*^+/+^ and *Snap25*^Δ3/Δ3^ mice immediately upon stimulation, 1 second after the stimulus, and at the peak of the stimulus. (**D**) Decay times in seconds from peak to baseline per field. (**E**) Bar graphs of the peak area of responding puncta (in μm^2^). *n* = 5 *Snap25*^+/+^ mice; *n* = 3 *Snap25*^Δ3/Δ3^ biological replicates, with all puncta displayed on the graphs. *P < 0.05 and **P < 0.01. Values are displayed as box-and-whisker plots, with a plus sign representing the mean. All analyses were performed using an unpaired, 2-tailed Student’s *t* test.

To directly test the hypothesis that removing Gβγ inhibition of SNARE-mediated vesicular fusion causes more NE to be secreted when sympathetic neurons are excited, we measured NE release from electrode-stimulated sympathetic nerves innervating iWAT using a selective, genetically encoded NE biosensor, GRAB_NE_ (38). This allows for good spatiotemporal resolution of junctional NE secretion. GRAB_NE_ expression was placed under the control of *TH*-Cre, which limits its expression to catecholaminergic and dopaminergic neurons. Individual neuronal processes adjacent to adipocytes from excised iWAT were imaged using lattice light sheet fluorescence microscopy (Supplemental Figure 6, A and B) (39–41). When exogenous NE was applied to sympathetic neurons innervating iWAT, fluorescence was stimulated in GRAB_NE_-expressing neurons (Supplemental Figure 6C). Plotting the fluorescence over time showed that GRAB_NE_ was rapidly excited by NE (Supplemental Figure 6D). High concentrations of epinephrine stimulated GRAB_NE_, but to a much lesser extent, demonstrating the fidelity of GRAB_NE_ for NE (Supplemental Figure 6E). Electrode stimulation of sympathetic nerves with a train of stimuli led to repeatable transient fluorescent puncta on sympathetic nerve endings (Figure 11A). GRAB_NE_ fluorescence was localized to the rim adjacent to the round cell bodies of adipocytes.

We used this system to compare the NE release profile between iWAT sympathetic neurons from *Snap25*^+/+^ and *Snap25*^Δ3/Δ3^ mice (Figure 11B). The initial stimulation of sympathetic neuronal axons produced a similar response (Figure 11C). At both 1 second after stimulation and during the maximal response, the stimulus-evoked GRAB_NE_ fluorescence in sympathetic neurons from *Snap25*^Δ3/Δ3^ mice was significantly greater (Figure 11C). The time required for fluorescence to return to baseline was also greater in *Snap25*^Δ3/Δ3^ mice (Figure 11D). Additionally, *Snap25*^Δ3/Δ3^ mice had a greater area of fluorescence response, consistent with the idea that these mice have more iWAT sympathetic innervation (Figure 11E). These findings are consistent with our prediction that the SNAP25Δ3 mutation removes a brake on NE exocytosis without affecting the initial exocytosis event. In summary, in this experiment we used electrical stimulation to directly provoke NE secretion from sympathetic nerves in iWAT. This allowed us to specifically measure the NE release profile from iWAT sympathetic nerves, which in *Snap25*^Δ3/Δ3^ mice we found to be increased in both amplitude and duration. This is the likely mechanism by which mild cold stimulation of the sympathetic nervous system caused by room temperature housing led to the large increase in iWAT NE content and UCP1-positive adipocytes in *Snap25*^Δ3/Δ3^ mice.

**Figure 12:**
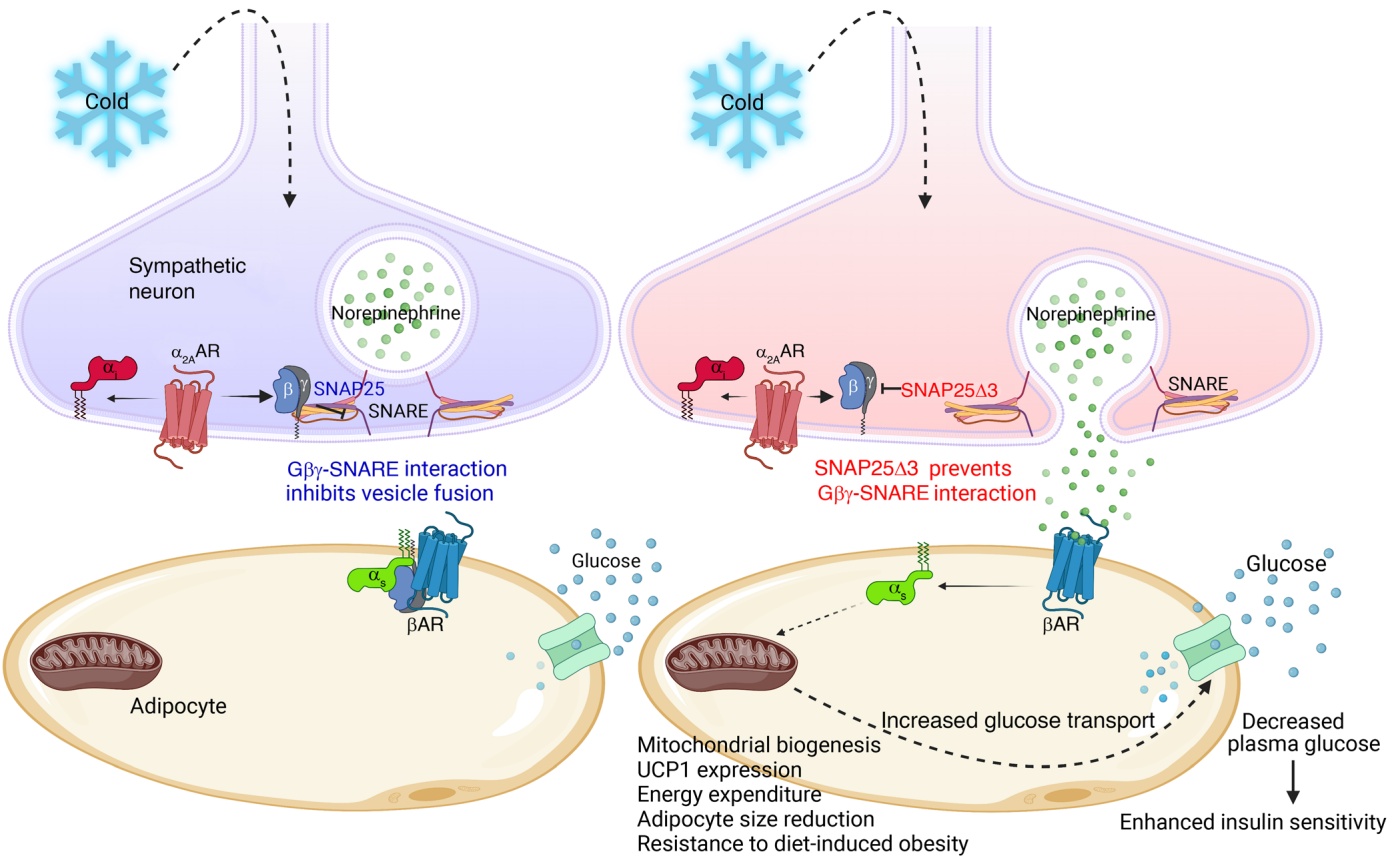
Summary of how removing the Gβγ-SNAP25 brake on exocytosis promotes adipocyte browning and glucose clearance. During cold stress, sympathetic regulation of adipocyte beiging/browning occurs through release of NE from sympathetic neurons and thereby induces the adipocyte β-adrenergic program. This increases mitochondrial biogenesis (browning) and expression of UCP1, generating heat by expending energy through uncoupled respiration. Housing mice at room temperature (normal housing is around 22°C) evokes a mild cold stress that is generally unnoticed. Normally there is an α_2_-adrenergic brake on neurotransmitter vesicle fusion that works through the Gβγ-SNARE interaction to limit NE release. We removed this brake on NE release by creating a mutant SNAP25 that decreases Gβγ binding, leading to enhanced and prolonged NE release from sympathetic neurons and, thereby, greater induction of the adipocyte thermogenic program. This enhanced browning increases glucose uptake into beige adipocytes, resulting in enhanced insulin sensitivity, particularly in these white adipose depots. We posit that this global genetic alteration enhances and prolongs synaptic vesicle exocytosis from SNAP25-expressing neurons associated other organ systems, such as those responsible for food intake. A result of these adaptations, *Snap25*^Δ3/Δ3^ mice are resistant to diet-induced obesity. Removal of cold-induced sympathetic activity by placing animals under thermoneutral conditions (~30°C) reverses all of these changes, allowing us to conclude that the inability of Gβγ to act as a negative regulator of NE release is the main driver of the beneficial metabolic phenotype.

We posit that the fundamental process driving the phenotype of this mouse lacking the Gβγ brake on SNARE-mediated vesicle fusion is amplified neurotransmitter release from the sympathetic nervous system. This was most apparent in the exacerbated sympathetic response to the mild cold stress caused by housing mice at room temperature, which enhanced the release of NE, leading to induction of the adipocyte thermogenic program (Figure 12). This increased sympathetic activity likely affected other metabolically active organs. NE induction of adipose tissue beiging and the thermogenic program in *Snap25*^Δ3/Δ3^ mice likely contributed to improvements in insulin action, particularly in adipose tissue. This increased sympathetic neural drive caused *Snap25*^Δ3/Δ3^ mice, which only lack the inhibitory actions of Gβγ on the SNARE complex, to be remarkably resistant to diet-induced obesity and the associated impairment in glycemic control.

## Discussion

We began these studies to examine the role of the Gβγ-SNARE interaction in the fusion and secretion of insulin-containing vesicles. Although SNAP25 is primarily expressed in neural cell populations (42, 43), it is also highly expressed in the β cells of pancreatic islets (44, 45). Prior studies using an immortalized β cell line showed that inhibition of insulin secretion by the α_2_AR autoreceptor occurs in part through Gβγ-SNARE interaction (13). Our *Snap25*^Δ3/Δ3^ mice provided an opportunity to test these findings both ex vivo, using intact islets, and in vivo, by measuring circulating insulin in response to a glucose challenge. However, we found no evidence that this negative regulation of insulin secretion by the α_2_AR was removed in *Snap25*^Δ3/Δ3^ mice, although this could be explained by differences between isolated ex vivo islets and the cultured β cell line. Another possibility is that in islet β cells, a protein other than SNAP25 provides the scaffolding for the Gβγ interaction with insulin granules (46–48), such as SNAP23 (49). During the course of these studies, we found that lean, chow-fed *Snap25*^Δ3/Δ3^ mice did not secrete as much insulin, although glucose levels were similar during the GTTs. This indicates that insulin-dependent glucose disposal was enhanced. Studies utilizing glucose clamps confirmed that insulin action was improved in *Snap25*^Δ3/Δ3^ mice. This compelled us to conduct a series of studies to determine why these *Snap25*^Δ3/Δ3^ mice would have improved insulin sensitivity.

Our first clue to the mechanism behind these phenotypes came from the observation that *Snap25*^Δ3/Δ3^ mice had increased beiging of their iWAT. This was particularly noticeable in *Snap25*^Δ3/Δ3^ mice challenged with a HFD, since the histology revealed extensive patches of beige adipocytes in iWAT. This partially explains the improved insulin sensitivity observed in *Snap25*^Δ3/Δ3^ mice, as the glucose tracer studies showed increased uptake in iWAT. However, it should also be noted that glucose uptake in iWAT may be driven by GLUT1 expression in beige adipocytes (50). We did not observe any notable increase in energy expenditure due to adipocyte beiging in *Snap25*^Δ3/Δ3^ mice. Therefore, the real benefit of beiging in this context appears to be enhancement of insulin-stimulated glucose uptake in adipose and other tissues such as liver and muscle (51, 52). Although iWAT beiging may not account for the largest proportion of the change in glucose uptake in *Snap25*^Δ3/Δ3^ mice, it was a notable contributor to the improvement in insulin action and was very striking.

A substantial number of hormones are known to influence the adipocyte thermogenic program, many of them through GPCR-mediated signaling events, but these are mediated by the α-subunits and their effects on cAMP/PKA signaling pathways (6). This fact, and reports that SNAP25 is not expressed in adipocytes (53–56), led us to suspect that the iWAT beiging phenotype was not due to the actions of Gβγ-SNARE in the adipocytes themselves, but rather of some hormone that is dysregulated in *Snap25*^Δ3/Δ3^ mice. We initially suspected sympathetic signaling because *Snap25*^Δ3/Δ3^ mice have an enhanced thermogenic response to handling stress (16, 25), but this was discounted after we failed to observe a differential response with a 6°C cold challenge or in circulating catecholamine levels.

We sought to come up with a way to effectively “turn off” the actions of the SNAP25Δ3 mutation. Our prior work shows that *Snap25*^Δ3/Δ3^ mice have an exacerbated response to stress (16, 25). It is known that room temperature housing is a mild cold stress for mice (57, 58) and that cold stress can induce WAT beiging (59). So, we hypothesized that a heightened response even to moderate cold stress could account for some of the phenotypic differences observed in *Snap25*^Δ3/Δ3^ mice. When housed under thermoneutral conditions, the metabolic phenotype of *Snap25*^Δ3/Δ3^ mice was completely normalized. This strongly indicated that sympathetic activity was altered in room temperature–housed *Snap25*^Δ3/Δ3^ mice, so we revisited the idea that NE levels might be different when measured in frozen iWAT samples. We found an enormous 23-fold increase in NE levels in the room temperature–housed mice, which completely returned to baseline in samples from mice housed under thermoneutral conditions. Because all metabolic differences were normalized by thermoneutral housing, and these differences cannot be accounted for solely by the lack of iWAT beiging, we posit that autonomic activity is increased in room temperature–housed *Snap25*^Δ3/Δ3^ mice, driving the metabolic protection.

We can infer from the studies discussed thus far that in iWAT, removing this Gβγ brake amplified the cold-stimulated NE secretion. We previously showed that α_2A_ARs work through Gβγ-SNARE interactions (16). To directly test this mechanism, we used the GRAB_NE_ fluorescent NE biosensor to unambiguously measure the NE release profile from iWAT sympathetic neurons in these mice (38). Direct stimulation of sympathetic nerves in iWAT in *Snap25*^Δ3/Δ3^ mice did not affect the initial NE exocytosis but rather intensified and prolonged the NE release. The effect of the *Snap25* genotype on the timing of the NE release profile is consistent with a model of defective α_2A_AR autoreceptor signaling, in which junctional NE must first rise to exert a negative feedback. We posit that loss of the brake to SNARE-mediated vesicle fusion and neurotransmitter release enhances the net release of NE, leading to increased autonomic activity.

The metabolic phenotype of the *Snap25*^Δ3/Δ3^ mouse is more complex than just WAT beiging. The altered feeding behavior of *Snap25*^Δ3/Δ3^ mice appeared to be a major contributor to their resistance to diet-induced obesity. Upon calculation of the net energy balance (total energy expenditure – caloric intake), *Snap25*^+/+^ mice tended to remain in a more positive energy balance than did *Snap25*^Δ3/Δ3^ mice during HFD feeding. This initial relative hypophagia in *Snap25*^Δ3/Δ3^ mice was likely a major contributor to their resistance to HFD-induced obesity, as we observed no differences in fasting-induced hyperphagia or food preference. The fact that differences in food intake were reversed at thermoneutrality suggests that this was also controlled by autonomic activity. This finding might be predicted, since environmental temperature and the sympathetic nervous system are known to affect feeding behavior (60–62). Given the impact of central autonomic drive for the modulation of food intake and energy expenditure, future studies could target specific brain regions to integrate this Gβγ-SNARE mechanism into our current understanding of the complex circuitry controlling appetite.

In summary, we found that disabling the Gβγ-SNARE brake on exocytosis in mice in vivo caused an exacerbation of mild cold stress from room temperature housing, leading to elevated autonomic NE release. Our findings parallel those of a recent study using a brain-sparing sympathofacilitator (PEGylated amphetamine) to increase autonomic activity, which showed increases in WAT NE levels and resistance to diet-induced obesity (63). Retrospectively, it makes sense that mice given amphetamines, which inhibit the reuptake of monoamine neurotransmitters, and the *Snap25*^Δ3/Δ3^ mouse would have phenotypic similarities, such as reduced food intake, increased iWAT beiging, and resistance to diet-induced obesity. These studies highlight the importance of considering changes in the sympathetic nervous system when evaluating metabolic phenotypes, as even slight alterations in autonomic activity can have profound physiological consequences. Our findings also demonstrate how chronic thermal stress due to housing mice at room temperature can have a drastic effect on phenotype. These studies present a striking example of an interaction between genotype and thermal environment. Mechanistically, we clearly show that disabling the Gβγ-SNARE interaction in sympathetic neurons innervating iWAT enhanced and prolonged NE release upon stimulation. Pharmacological targeting of this Gβγ-SNARE interaction could be an interesting alternative to direct targeting of the GPCRs themselves, given the broad improvements in food intake, insulin action, and adipose browning, which all occurred without cardiovascular effects. Any potential neurological side effects could be ameliorated through the design of CNS-sparing agents, while avoiding GPCR targets that could have broad tissue distributions and side effects. Thus, we believe that targeting a pathway that modulates, rather than drives, specific neuroendocrine signals is a unique approach to treat metabolic disease.

## Methods

### Animal procedures.

Mice were generated through heterozygous breeding of *Snap25*^+/Δ3^ mice on a C57BL/6 background as described previously (16) and were housed at Vanderbilt University unless otherwise stated. Mice were maintained on 12-hour light/12-hour dark cycles with 3–5 animals per cage except during food intake monitoring, at which times they were singly housed. Mice were housed at standard housing room temperature (~22°C) or in thermoneutral housing conditions (~29.5°C) when indicated. Mice were maintained ad libitum on chow (13.5% calories from fat: 5001; LabDiet) or a HFD (60% calories from fat; 3282, Bio-Serv) when indicated. Weights and body composition measurements using the LF50 Body Composition Analyzer (Bruker) were done at the Vanderbilt Mouse Metabolic Phenotyping Center (VMMPC). All studies were performed with littermates of the appropriate genotypes. Mice were fasted for 5 hours and then euthanized by isoflurane overdose or CO_2_ asphyxiation and exsanguination via cardiac puncture at the end of the study for collection of blood and tissues. Data from one mouse was excluded from all studies due to hydronephrosis.

### Histology.

Tissues were fixed in 10% formaldehyde overnight and subsequently stored in 70% ethanol and routinely processed. The tissues were then embedded, sectioned, and stained with H&E or were immunohistochemically stained for TH (ab152, MilliporeSigma) or uncoupling protein 1 (UCP1) (ab10983, Abcam). Histological analysis was performed by the Vanderbilt Translational Pathology Shared Resource. Whole slides were imaged at ×20 magnification with a Leica SCN400 Slide Scanner by the Digital Histology Shared Resource at Vanderbilt University Medical Center.

Quantification of TH staining was performed in QuPath, version 0.3.0. One whole section each of iWAT and iBAT was quantified for each mouse. Blood vessels, lymph nodes, and dissection artifacts were manually excluded from analysis. For all slides, the hematoxylin stain was set at 0.63_0.682_0.371, and the TH stain was set at 0.479_0.618_0.623. Counting was performed using the Fast Cell Counts (bright-field) feature. The cell detection threshold was set at 0.3, and the DAB threshold was set at 0.6. All other parameters were the default values.

Quantification of iWAT adipocyte cell size was performed in Fiji adapted from a method developed by Joseph Roland (https://www.vumc.org/dhsr/sites/default/files/public_files/Fiji-Adipose-Segmentation.pdf). Briefly, a TIFF image of the entire iWAT section at a resolution of 0.5 μm per pixel was uploaded into Fiji, and the area outside of the tissue was manually cropped out. The image was converted to 8 bit, and the colors were inverted. The background was subtracted using the settings of Rolling Ball Radius 20.0 pixels and Sliding Paraboloid. The image was manually cropped into multiple segments to reduce file size. Morphological Segmentation with Gaussian radius 3 and Watershed Segmentation tolerance 4 was run on each segment. An image was created showing the dams from the Morphological Segmentation, and a Gaussian blur with a Sigma of 2.0 was added. The resulting image was converted to 8 bit, and the threshold settings were manually adjusted so that the cell walls were white on a black background. Analyze Particles was run with the size range of 500-infinity, producing a list of adipocyte areas in pixels. The area was converted to μm^2^, and measurements below 125 μm^2^ and above 25,000 μm^2^ were excluded. All adipocytes size measurements within this range were plotted for each individual mouse, with 25,463–65,480 measurements per mouse. Analyses comparing genotypes were performed using the median adipocyte size.

### Leptin.

At the end of the chow- or HFD-feeding studies, mice were euthanized as described above, and blood was obtained postmortem by terminal cardiac puncture and placed into a microcentrifuge tube coated with EDTA. Samples were kept on ice until centrifugation to obtain plasma, which was stored at –80°C. Leptin was measured from this plasma with a Luminex 100 system at the Vanderbilt University Hormone Assay and Analytical Services Core.

### GTT.

For all GTTs, mice were fasted for 5 hours, and fasting blood glucose was measured from a drop of tail vein blood (Accu-Chek Aviva glucometer) at the indicated time points. GTTs in Figure 9 were conducted in a 29.5°C room, while all other GTTs were conducted at standard room temperature. For Figure 2, glucose was given by either an intraperitoneal injection or oral gavage of 2.0 g/kg glucose. For Figures 5 and 9, mice were given intraperitoneal injections of 1.0 g/kg dextrose (Agri Laboratories) in 0.9% saline (Hospira). Insulin concentrations during GTTs (Figures 2 and 9) were analyzed by RIA at the Vanderbilt University Hormone Assay and Analytical Services Core. In Figure 5B, mice were fasted for 5 hours, and blood glucose was collected from the tail vein, after which the mice were euthanized by CO_2_, and EDTA plasma was collected by cardiac puncture as described above, which was stored at –80°C until insulin was measured by ELISA (Mercodia).

### Mouse islet perifusion.

Pancreatic islets were isolated, and perifusion assays were performed on fresh islets at the Vanderbilt Islet Procurement and Analysis Core as previously described (64). Islet preparations were equilibrated, a stable baseline response was established at 5.6 mmol/L glucose, and insulin secretion was stimulated with 16.7 mmol/L glucose. A dose-response curve for the inhibition of GSIS by the α_2_AR selective agonist Br was generated. Islets were matched for size and number and assessed as islet equivalents (IEQs) (65).

### Energy balance.

Food intake and energy expenditure were monitored in mice by the VMMPC using a Promethion system (Sable Systems International). Animals were housed over multiple days to acclimate to the facility. Body weight and composition were monitored before and after a calorimetry study.

### Cardiovascular imaging.

Cardiac parameters were measured by parasternal M-mode echocardiography at the VUMC Cardiovascular Physiology Core as described previously (66, 67). Briefly, the chest fur of the mice was removed with a topical depilatory agent. Ultrasound coupling gel heated to 34°C was applied to the chest area, and a linear array transducer (18–23 MHz) was positioned to obtain 2-dimensional B-mode parasternal long- and short-axis views at the mid-ventricular level (Vevo 2100, VisualSonics). One-dimensional M-mode images were taken for measurement in the short-axis view to determine cardiac wall thickness and chamber dimensions. Left ventricular (LV) chamber size and wall thickness were measured off-line in the M-mode from at least 3 consecutive beats and averaged. LV wall thickness (interventricular septum [IVS] and posterior wall [PW]) at systole and diastole and LV internal dimension (LVID) during systole and diastole were measured”

### Hyperinsulinemic euglycemic clamps.

Clamp studies were performed in chronically catheterized (carotid artery and jugular vein) conscious mice (68–70). Catheters were inserted 4–5 days prior to a study by the VMMPC. [3-^3^H] glucose was used to measure whole-body basal and clamp glucose flux. A 4 mU/kg/min insulin infusion was initiated to increase insulin to a physiologic range. RBCs from a donor animal were infused at a constant rate to replace blood taken during the study. Basal and clamp period blood samples were obtained for glucose, insulin, and tracer evaluation. At the end of the clamp period, multiple tissues were collected to measure the accumulation of 2-DG. Using tracer methods [3-^3^H]-glucose and 2-DG during the clamp, we assessed tissue glucose uptake and whole-body (and hepatic) glucose flux.

### Catecholamines.

To minimize stress-induced catecholamine release, mice were cannulated and allowed to move about freely while blood was collected using a blunt needle. Cannulations and blood collections were performed by the Vanderbilt Brain Institute Neurochemistry Core. For catecholamines in adipose tissue, whole flash-frozen iWAT was ground into a powder in liquid nitrogen. Powdered iWAT was poured into a tube and weighed. PCA solution (5 mM glutathione in 0.4 N perchloric acid) was added to frozen iWAT powder at a ratio of 1 mL PCA/100 mg iWAT. Samples were homogenized and then centrifuged. The clear supernatant was removed and stored at –80°C. Catecholamines in both plasma and frozen tissue lysates were analyzed by liquid chromatography/mass spectrometry (LC/MS) and HPLC, respectively, with electrochemical detection as previously described (71) at the Vanderbilt Hormone Assay and Analytical Services Core.

### Fecal triglycerides.

Feces were collected from the cages of mice that had been fed a HFD for 8 weeks, and triglyceride levels were quantified by the VUMC Lipid Core.

### Feeding behavior studies.

For weekly food intake studies, individually housed animals were given ad libitum access to a preweighed amount of food that was weighed again at the end of each week. For fasting-refeeding studies, individually housed chow-fed mice were fasted for 12 hours before being given ad libitum access to a preweighed amount of the HFD food, which was weighed hourly. For food preference studies, individually housed chow-fed mice were given ad libitum access to preweighed amounts of both chow and a HFD. At the end of 7 days, chow and the HFD food were weighed again. Studies were conducted at room temperature unless otherwise stated.

### Imaging of GRAB_NE_ fluorescence transients in iWAT.

*Snap25*^Δ3/Δ3^ mice or *Snap25*^+/+^ littermates hemizygous for *Rosa26-LSL-jRGECO1A-2A-GRAB-NE* (gift from Yulong Li, Peking University, Beijing, China) and *TH*-Cre [B6.Cg-7630403G23RikTg(Th-cre)1Tmd/J, JAX] housed at the University of Illinois at Chicago were anesthetized with isoflurane and sacrificed via cervical dislocation. GRAB_NE_ was expressed selectively on catecholaminergic neurons with *TH*-Cre. Incisions were made dorsal-ventral at the axilla and rostral-caudal down the length of the spine. Fascia were transected and iWAT was immediately cut from connecting tissue and placed in a solution of 93 mM *N*-methyl-d-glucamine, 2.5 mM KCl, 1.2 mM NaH_2_PO_4_, 20 mM HEPES, 10 mM MgSO_4_, 0.5 mM CaCl_2_, 25 mM d-glucose, 5 mM ascorbate, and 3 mM pyruvate, bubbled with 95% O_2_ and 5% CO_2_, pH7.4. iWAT was mounted with small pins through the lateral edges to a silicone elastomer stage and imaged via lattice light sheet microscopy. The solution was exchanged for 124 mM NaCl, 26 mM NaHCO_3_, 1.25 mM NaH_2_PO_4_, 3 mM KCl, 2 mM CaCl_2_, 1 mM MgCl_2_, and 10 mM d-glucose, bubbled with 95% O_2_ and 5% CO_2_. A twisted-pair stimulating electrode was placed on adjacent ganglia from T11-L1, and GRAB-NE fluorescence transients (excitation 485 nm/emission 535 nm) were recorded using stimulus trains of 100 stimuli, 100–200 mA at 20 Hz, and 1 ms duration. Each image was captured with a 50 ms exposure time. Transient data were analyzed as the change in fluorescence intensity relative to the resting fluorescence intensity using ImageJ (NIH).

### Statistics.

Data represent the mean ± SEM, and statistical significance was determined using GraphPad Prism version 9.1.0(221) for Windows 64-bit (GraphPad Software). Analyses comparing genotypes only were performed with an unpaired, 2-tailed Student’s *t* test. Analyses comparing 2 independent variables were performed with a 2-way ANOVA or a mixed-effects model if data were missing. Multiple-comparison tests were performed with Bonferroni’s correction for the *Snap25* genotype only. Whole-body energy expenditure data were analyzed using analysis of covariance (ANCOVA) (72, 73). A *P* value of less than 0.05 was considered significant.

### Study approval.

The animal protocols were approved by the Vanderbilt University (Nashville) and University of Illinois at Chicago IACUCs.

### Data availability.

All underlying data can be found in the Supplemental Supporting Data Values file.

## Author contributions

ZZ conceived the initial idea for the *Snap25*^Δ3/Δ3^ mouse and insulin secretion studies, designed and led the early research and GRAB_NE_ studies, conducted experiments, analyzed data, and contributed to writing of the manuscript. RPC designed and led subsequent research studies centered on adipose tissue browning, conducted experiments, analyzed and organized data, wrote the manuscript, and created the figures. RPC is listed first because he initiated the investigation into the adipocyte browning, led these subsequent studies, and oversaw the final version of the manuscript and figures. ATG managed the mouse colony, conducted experiments and acquired, analyzed, and managed the data. FA helped maintain the mouse colony and assisted with mouse studies. AMB conducted the adipocyte size analysis. FS aided in the collection of mouse tissues. DL aided in the collection of mouse tissues and performed Western blotting. JM provided helpful insight into adipocyte innervation. JF and YL provided the GRAB_NE_ mice. SA designed and oversaw the studies to measure NE release using GRAB_NE_ mice. JEA designed food intake and metabolic cage studies, analyzed data, and contributed to writing of the manuscript. OPM designed experiments, analyzed data, and wrote the manuscript. SC designed experiments and wrote the manuscript. HH designed experiments, wrote and organized the manuscript, and was responsible for the study overall.

## Supplementary Material

Supplemental data

Supporting data values

## Version 1 08/10/2023

In-Press Preview

## Version 2 10/02/2023

Electronic publication
